# Raman Sensitive Degradation and Etching Dynamics of Exfoliated Black Phosphorus

**DOI:** 10.1038/srep44540

**Published:** 2017-03-20

**Authors:** Fadhel Alsaffar, Sarah Alodan, Abdul Alrasheed, Abdulrahman Alhussain, Noura Alrubaiq, Ahmad Abbas, Moh. R. Amer

**Affiliations:** 1 Center of Excellence for Green Nanotechnologies, Joint Centers of Excellence Program King Abdulaziz City for Science and Technology P.O Box 6086, Riyadh 11442, Saudi Arabia; 2 Department of Electrical Engineering 3740 McClintock Avenue University of Southern California, Los Angeles, CA, 90089, USA; 3 Department of Electrical and Computer Engineering University of Jeddah, 285 Dhahban 23881, Saudi Arabia; 4 King Abdulaziz University, Abdullah Sulayman Street, Jeddah, 22254, Saudi Arabia; 5 Department of Electrical Engineering 420 Westwood Plaza, 5412 Boelter Hall University of California, Los Angeles, CA, 90095, USA

## Abstract

Layered black phosphorus has drawn much attention due to the existence of a band gap compared to the widely known graphene. However, environmental stability of black phosphorus is still a major issue, which hinders the realization of practical device applications. Here, we spatially Raman map exfoliated black phosphorus using confocal fast-scanning technique at different time intervals. We observe a Raman intensity modulation for 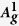, ***B***_**2*****g***_, and 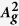 modes. This Raman modulation is found to be caused by optical interference, which gives insights into the oxidation mechanism. Finally, we examine the fabrication compatible PMMA coating as a viable passivation layer. Our measurements indicate that PMMA passivated black phosphorus thin film flakes can stay pristine for a period of 19 days when left in a dark environment, allowing sufficient time for further nanofabrication processing. Our results shed light on black phosphorus degradation which can aid future passivation methods.

Newly discovered black phosphorus (B.P.) has been the focus of recent research for various device applications. Owning to the existence of a thickness dependent band gap^1^, high carrier mobility^2^,^3^, strong exciton binding energies^4^, and extraordinary thermoelectric properties^5^,^6^, layered black phosphorus has proven to be a potential candidate for device applications. Various reports have shown exceptional optical properties, optoelectronic properties, and photoresponse performance of black phosphorus thin film devices^7^,^8^,^9^,^10^,^11^,^12^,^13^,^14^,^15^. However, a major obstacle facing black phosphorus devices is the noticeable fast photo-reaction to ambient environment, which forms oxidized black phosphorus, resulting in degraded device performance. To date, there have been few reports on black phosphorus degradation and encapsulation studies^16^,^17^,^18^,^19^,^20^,^21^,^22^. Most notably, Favron *et al*. studied the degradation mechanism in few layers black phosphorus^23^. Their results indicate phosphorene degrades in a short period of time, evident by the exponential decay in the intensity of each Raman mode. Island *et al*. measured the instability of black phosphorus^24^ using AFM measurements. They found that the volume of phosphorene increases with time. They also reported etching of phosphorene layers accompanied the degradation process, which can be observed in the decreasing conductance of black phosphorus FET device. Moreover, attempts to prevent oxidation has also been reported with different passivation techniques. Wood *et al*. showed that Al_2_O_3_ passivation is an effective method to prevent oxidation from ambient environment, preserving black phosphorus FET characteristics for a period of 2 weeks^25^. Another recent study shows that coating black phosphorus with trilayer graphene is required to prevent oxidation^26^. Nevertheless, the technique lacks practicality for large scale applications.

In this work, we optically characterize the effect of ambient conditions on exfoliated black phosphorus flakes (*d* < 150 nm) using fast-scanning confocal μ-Raman spectroscopy. We show black phosphorus exhibit a unique degradation processes, which can be directly observed in the giant modulation of the Raman intensities for each vibrational mode. We also explore the origin behind this Raman intensity modulation and extract the etching dynamics with time (i.e. thickness, etching rate). Finally, we examine the effectiveness of the widely-used photolithography compatible Polymethyl methacrylate (PMMA) polymer as a viable passivation layer in order to assess the allowed time for further lithography processing while preserving the pristine nature.

## Results

### Fast-scanning confocal μ-Raman spectroscopy of layered black phosphorus

**Figure 1:**
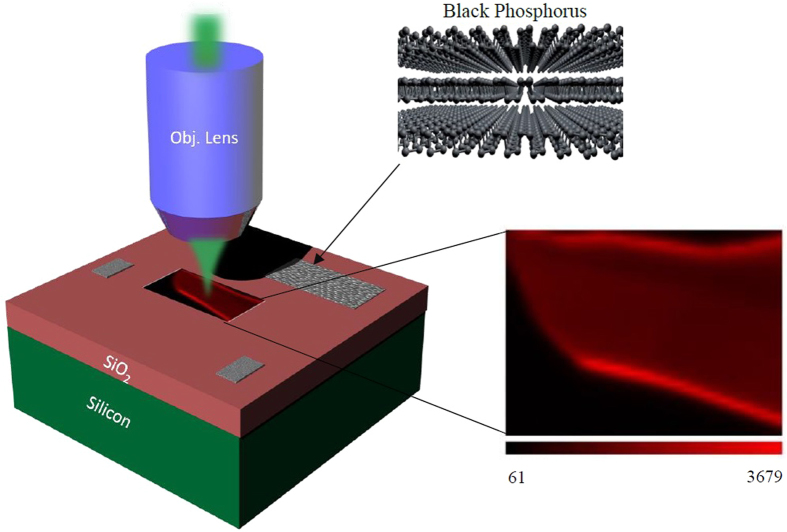
μ-Raman spectroscopy of exfoliated black phosphorus. (**a**) Schematics of fast-scanning confocal μ-Raman spectroscopy of layered black phosphorus. The image on the right corresponds to the measured Raman intensity map for *B*_2*g*_ peak.

Due to the high in-plane anisotropy of black phosphorus, the intensities of 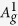 and 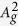 vibrational modes are angle dependent^27^. A schematic representation of the experiment and the optical setup details of layered black phosphorus is illustrated in Fig. 1 and S1, respectively. In the setup, the laser is incident on the sample which is positioned on a high-speed stage. The Raman scattered light is detected using a fast CCD detector. This setup enables us to scan a large area on the sample in a short time. In order to construct a μ-Raman intensity image of black phosphorus, a short exposure time with low laser power are implemented.

**Figure 2:**
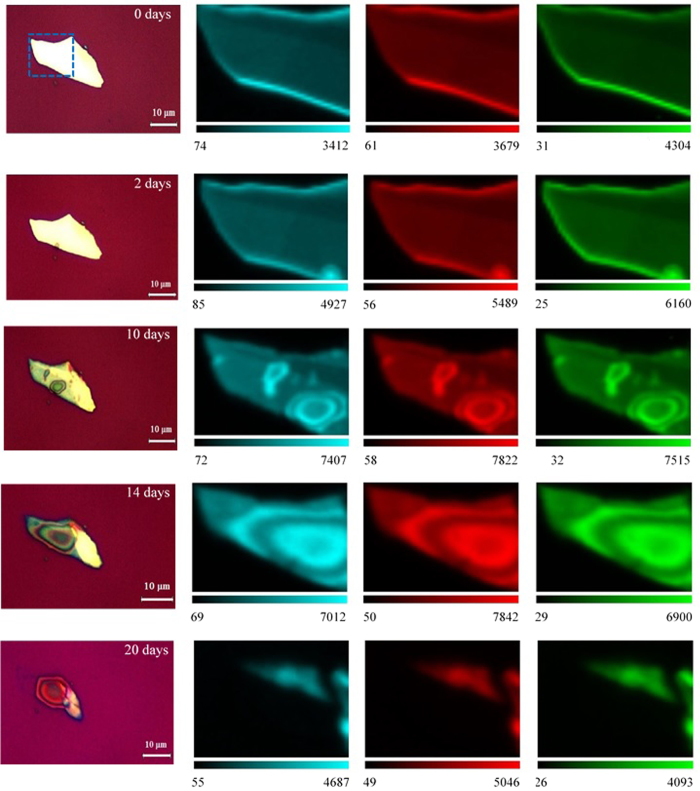
Spatial Raman measurements of exfoliated black phosphorus. Optical image and the corresponding Raman intensity maps of 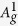 (cyan color), *B*_2*g*_ (red color), and 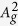 (green color) vibrational peaks at (**a**) as exfoliated (*t* = 0 days). (**b**) *t* = 2 days. (**c**) *t* = 10 days. (**d**) *t* = 14 days. (**e**) *t* = 20 days. The highlighted area in the optical image in (**a**) corresponds to the scanned area of interest. Sample thickness *d* = 35 ± 5 nm.

In Fig. 2, the measured optical images along with the measured Raman intensity maps of 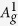, *B*_2*g*_, and 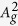 for a black phosphorus thin film flake (*d* = 35 ± 5 nm) are presented at different time intervals. For as exfoliated Raman maps, no degradation is observed, although a slight increase in the Raman intensities for all vibrational modes is shown at the edges of the flake. However, by the 2^nd^ day, a small circularly shaped degraded area is observed at the bottom edge of the flake. The Raman intensities at this area is significantly larger than the intensities of the neighboring areas, including the measured Raman intensities in prior days. On the 6^th^ day (Figure S1), although the optical image does not show visible signs of degradation on the surface of the flake, the measured Raman intensity maps show small degraded areas on the surface with intensity increase for each Raman mode. This intensity change, however, is lower than the prominent degraded area at the bottom edge. After *t* = 10 days (Fig. 2c), the surface starts to exhibit significant degradation, while the degraded area at the bottom edge starts to expand rapidly. The measured Raman intensities of both degraded areas show similar intensities with magnitudes much higher than neighboring areas. After 14 days, both areas expand and merge together to form a larger circularly shaped degraded area. For each Raman mode, the circular shape is reflected in the different intensities observed on the surface of the flake (Fig. 2d). The observed Raman intensity maps for the successive days show similar profile until the flake is completely etched. This degradation trend is also confirmed on different samples with similar thickness (see Figures S8–S10 in the supplementary document for more information).

### Large Raman intensity modulation

**Figure 3:**
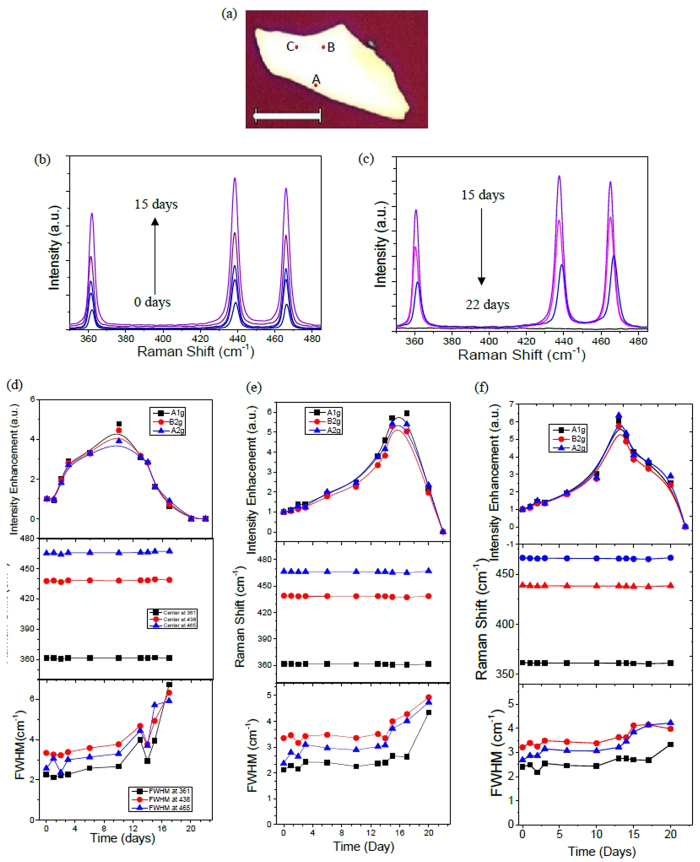
Raman characteristics of exfoliated black phosphorus. (**a**) optical image showing sites A, B, and C. The scale bar is 10 μm. (**b**) and (**c**) are Raman spectra at different time intervals showing the Raman intensity modulation for site B highlighted in (**a**). The Raman intensity, Raman shift, and FWHM for (**d**) site A and (**e**) site B, and (**f**) site C. Sample thickness *d* = 35 ± 5 nm.

In order to understand this Raman intensity modulation with degradation, we plot in Fig. 3 the Raman intensities at 3 different locations as a function of time for each Raman mode. Site A is near the edge of the flake and corresponds to the first observation of Raman intensity change, while site B and C are near the middle of the flake, as highlighted in Fig. 3a. We notice a considerable intensity enhancement for each of the vibrational peaks in the Raman spectra, which is plotted in Fig. 3b and c for site B. In Fig. 3d,e and f, the Raman characteristics for each site are plotted at different degradation stages. We first note that a rapid increase in the Raman intensities of 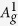, *B*_2*g*_, and 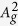 is observed for site A, which starts by *t* = 2 days and peaks on *t* = 10 days, followed by intensity drop. However, the Raman intensities for sites B and C show a gradual increase and peak on *t* = 15 days and *t* = 13 days, respectively. It is important to mention that we did not observe noticeable change in the Raman shift for each Raman mode in the initial days, however, before full degradation, the Raman shift started to show an upshift for each Raman mode as shown in Figure S3, which is consistent with previously reported shifts^23^,^28^. Other flakes have also shown this Raman upshift before complete degradation (see Figure S10 in the supplementary document). For all sites, the FWHM shows a slight increase at late stages of degradation until the flake is completely degraded.

In order to understand the dependence of this Raman intensity modulation on the flake thickness, we carried out Raman measurements on additional samples with different thicknesses, mainly few layers flake and thick thin film flake. For few layer flake (*d* ≈10 nm), the intensity of all Raman modes decrease as shown in Figures S5–S7, which is in good agreement with previous reports^18^,^23^. However, for thick flakes (*d* > 60 nm), a different trend is observed. The Raman intensity shows a monotonically decreasing oscillatory behavior, as illustrated in Figure S13 for a specific site on the thin film flake. We observe 3 different time intervals where the Raman intensity showed enhancement, followed by Raman intensity decrease. Our measurements suggest that this Raman intensity modulation depends on the layer thickness as black phosphorus degrades with time.

### Estimating the degradation etching Rate

**Figure 4:**
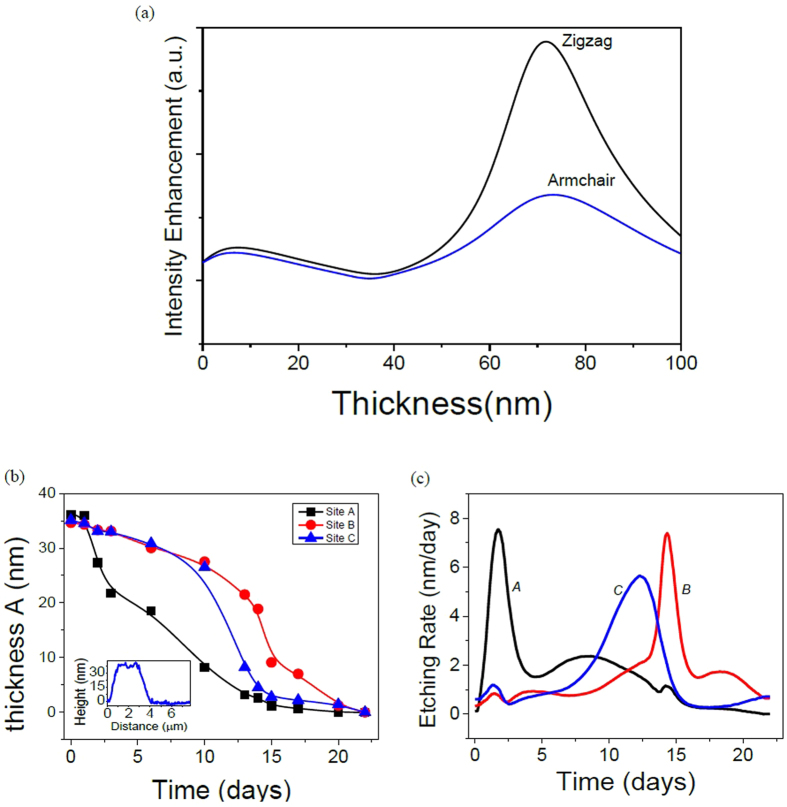
Optical interference and estimated thickness and etching rate at different time intervals. (**a**) calculated normalized intensity enhancement as a function of black phosphorus thickness for zigzag and armchair directions. (**b**) average thickness and (**c**) estimated etching rate vs. time for sites A (black square), B (red circle), and C (blue triangle). The inset in figure (**b**) is the measured AFM line profile of the initial thickness of the thin film flake. Sample thickness *d* = 35 ± 5 nm.

The Raman intensity modulation detected for 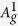, *B*_2*g*_, and 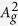 Raman modes can be explained by considering the optical interference at the interfaces (black phosphorus/SiO_2_/Si). In this model, the optical interference is caused by multiple Raman scattering events inside black phosphorus layers, along with multiple reflections of the incident laser beam (model schematics in Figure S16). This model has been previously used to explain the Raman intensity change in *G* and *D* Raman modes of few layers graphene compared to graphite^29^,^30^. In Fig. 4a, the calculated intensity enhancement of black phosphorus at different thicknesses is plotted for 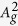 peak based on the intensity enhancement model. It should be noted that although the model depends on the Raman peak frequency, the calculated profile for each of 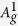, *B*_2*g*_, and 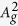 show miniscule differences (Figure S17). Moreover, it is possible that the Raman intensity enhancement calculations can be affected by the inclusion of PO_x_ layer. However, this layer exhibit refractive index close to air (*n* = 1.3) and it does not explain the oscillatory behavior in the Raman intensity vs. time measurements observed for different samples (see supplementary document for more data)^31^.

Previous reports have shown that the water droplets and liquid interfaces produce interference fringes on the surface of black phosphorus^25^,^32^, which could affect the observed Raman intensity modulation. Accordingly, we measured Raman intensity maps *in situ* with AFM measurements in order to identify the role of these liquid interfaces. Our AFM measurements show that major bubbles form and cover the entire degraded area, as shown in Figures S20 and S21. However, our Raman measurements of these degraded areas show intensity modulation maps where the intensity enhancement forms a ring-shaped profile near the edges of each bubble. We would expect the maximum intensity occurs in the middle (focal point) of the bubble if lensing effect was a factor in the Raman intensity enhancement. Nevertheless, we observe Raman intensity oscillations with increasing degradation time occurring underneath the bubble. We find that our intensity enhancement results originate from the degraded area underneath the bubble, which is due to phosphoric acid reactivity with the surface, resulting in non-uniform etching of the surface.

We also performed high temperature annealing (*T* > 100 °C) in vacuum in order to observe the possibility of intensity change after cleaning the surface. We measured the Raman intensity maps before and after annealing (Figure S22). Our measurements show that the Raman intensity slightly increases in the affected area after annealing for each Raman mode, which can be attributed to the removal of these liquid interfaces and localized etching of phosphoric acid to the top-most layer.

The optical interference can also explain the oscillatory behavior observed in Figure S13 for thick thin film flakes. The AFM measurements carried at a specific time interval shows a flake thickness of *d* = 55 ± 10 nm (Figure S14). The calculated non-normalized intensity enhancement due to optical interference shows that for a flake with this thickness, the intensity profile should exhibit a decrease followed by Raman intensity enhancement (Figure S17a). Indeed, our Raman intensity measurements show this profile, which is in good agreement with the optical interference model.

Using the interference model, one can estimate the thickness of the exfoliated black phosphorus at different time intervals for sites A, B, and C, by fitting the measured Raman intensity to the corresponding thickness (see supplementary document for details). The initial thickness of the black phosphorus flake is approximately *d* = 35 ± 5 nm as measured by AFM in Figure S4 (AFM line profile inset of Fig. 4b). For black phosphorus films with thicknesses between 0 nm–40 nm, the model exhibit similar profile for armchair and zigzag directions, which simplifies the extracted thickness vs. time calculations. Figure 3b shows the extracted average thickness as a function of time for sites A, B and C. The average thickness is obtained after extracting the thickness vs. time for each Raman mode. We should note that this thickness estimation vs. time relies on the initial thickness of black phosphorus which is measured using AFM. This method does not provide a new optical technique to identify the initial thickness of black phosphorus. This initial thickness is crucial to set the boundary conditions for the intensity enhancement model and estimate the thickness of black phosphorus at a certain time interval using the measured Raman spectra. For site A, the thickness exhibits a rapid decrease by the second day, while sites B and C show a steady decrease with time. Accordingly, we can estimate the etching rate at both of these sites. The etching rate (∂*d/*∂*t*) is essentially the differential form of the thickness (*d*) with respect to time (*t*). In Fig. 3c, the estimated etching rate for sites A, B, and C show a prominent peak at different time intervals. As expected, we observe a large etching rate for site A by *t* = 1.76 days compared to other sites. We also notice that the dominant peak profiles for sites A and B are analogous with a shift of Δ*t* = 12.24 days. For site C, however, a prominent broad peak at *t* = 12.3 days is observed. This different etching behavior for site C compared to sites A and B illustrates the different degradation processes black phosphorus exhibits, producing a non-uniform etching profile on the surface as discussed in details below.

### Polymethyl methacrylate (PMMA) passivation

**Figure 5:**
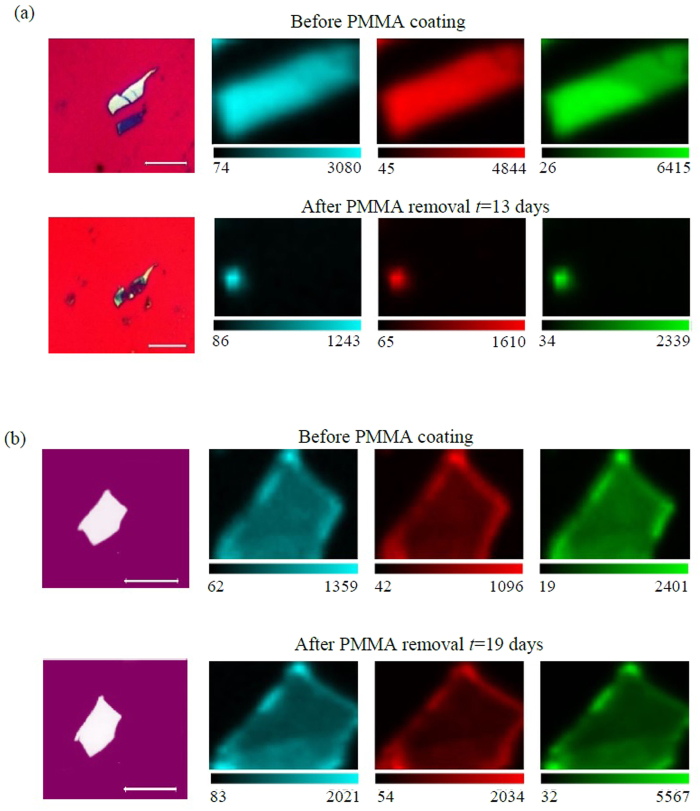
Spatial Raman measurements of PMMA coated black phosphorus. Optical image and Spatial Raman intensity maps of 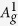 (cyan color), *B*_2*g*_ (red color), and 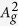 (green color) Raman modes for PMMA passivated (**a**) few layers black phosphorus (*d* < 10 nm), and (**b**) black phosphorus thin film flake (*d* < 60 nm). The scale bar in the optical images is 10 μm.

We explore the degradation of exfoliated black phosphorus when coated with PMMA. We choose PMMA as a passivation layer due to its transparency and nanofabrication compatibility, which allows further lithography processing on the flake. In Fig. 5a and b, the measured Raman intensity maps before and after PMMA coating are illustrated for 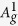, *B*_2*g*_, and 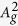 modes of few layers and thin film black phosphorus flakes. Each flake was coated with PMMA and left in dark environments for a period of 13 days and 19 days for few layers black phosphorus (*d* < 10 nm) and thin film black phosphorus flake (*d* < 60 nm), respectively. Raman intensity maps were taken before PMMA coating and after PMMA removal. For few layers black phosphorus, the flake degrades and disappears completely as seen in the optical image and confirmed with the Raman intensity measurements in Fig. 5a. However, for thin film black phosphorus flake, the optical image does not show any visible signs of degradation while Raman intensity maps before and after PMMA coating exhibit comparable results, suggesting minimal environmental effects on the flake. Accordingly, we deduce that PMMA coating of thin film black phosphorus gives sufficient period of time to perform further lithography processes.

In order to find the first stages of degradation of PMMA coated few layers black phosphorus flake, we measured the Raman intensity maps of PMMA coated few layers black phosphorus at different time intervals. In Figure S15, Raman intensity maps of each Raman mode show different degraded areas on the surface of the flake accompanied by a drop in the Raman intensities after *t* = 2days. In fact, the flake fully degrades by *t* = 5days as shown in Figure S8. These results indicate that a 2-day period is sufficient to perform further nanofabrication on PMMA coated few layers black phosphorus. However, caution should be taken into consideration due to the short period of time.

## Discussion

To this end, we discuss the degradation mechanism of black phosphorus thin film flakes. Based on our results obtained on different exfoliated black phosphorus samples, there are two competing processes of which oxygen reacts with layered black phosphorus, edge degradation and surface degradation. For thin film black phosphorus flake, edge degradation starts prior to surface degradation with a faster rate, evident by the change in the Raman intensities of each Raman mode. Surface degradation, on the other hand, shows a slower degradation rate compared to edge degradation. Eventually, both degradation processes expand and merge together to produce a non-uniform degraded surface.

The different etching rate profiles observed in the estimated etching rate for sites A and B are different from the etching rate for site C arise from the different degradation mechanisms discussed above. Site A is located near the edge where edge degradation process is first observed, while site B is located after the edge degradation process expands along the surface of the flake (see Figure S1c). However, site C is located near the surface degradation process that can be clearly seen in Fig. 2c. Accordingly, we infer that etching dynamics of black phosphorus thin film flakes in ambient conditions exhibit two different profiles which depends on the type of degradation mechanism mentioned above.

Few layers black phosphorus flakes, however, have a slightly different degradation mechanism. Due to its atomically thin nature, oxygen can react with the topmost layers as demonstrated in the spatial Raman intensity maps in Figures S5 and S6. In fact, unlike thin film flakes, few layers flakes disappear in hours scale, analogous to previously reported degradation measurements^23^. This difference in degradation mechanisms between few layers and thin film flakes arises from the different degradation kinetics discussed in ref. 23, which can be enhanced with quantum confinement resulting in faster degradation. A recent report also indicated that lowering of surface tension with decreasing thickness can accelerate the degradation mechanism^33^, and showed that few layers black phosphorus exhibit lower surface tension which can contribute to faster degradation process. Nevertheless, PMMA passivation can slow down the degradation process for a specific period of time and does not prevent degradation from occurring.

Unlike PMMA coated few layers black phosphorus, PMMA coating of thin film black phosphorus flakes has proven to be a good passivation method for a prolonged period of 19 days, provided that the substrate is left in a dark environment, according to our measurements. Such a simple method preserves the pristine nature of thin film black phosphorous flakes and make them attractive for future potential applications.

In conclusion, we have studied the degradation mechanism of black phosphorus using fast-scanning μ-Raman spectroscopy. We show a modulation in the measured Raman intensities for 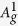, *B*_2*g*_, and 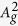 vibrational modes as a function of degradation time. This intensity modulation is found to be caused by optical interference inside black phosphorus thin film flakes due to the reduced thickness during degradation. Our results indicate that there are two degradation mechanisms where oxygen reacts with black phosphorus, edge degradation and surface degradation. Edge degradation dominates the degradation mechanism for thin film black phosphorus flakes, while surface degradation dominates the degradation mechanism of few layers black phosphorus. Using the Raman interference model, we can estimate the thickness and the etching rate due to oxidation at different sites on the black phosphorus flake. The calculated etching rate profiles at different sites on black phosphorus further confirm these competing degradation mechanisms. Finally, PMMA passivation is a viable encapsulation technique for a period of 19 days for black phosphorus thin film flakes. However, PMMA passivation of few layers can slow down the degradation for a short period of time (*t* = 2days) but suffers from ultimate flake degradation.

## Methods

### Sample preparation

black phosphorus flakes were prepared using micromechanical exfoliation with scotch tape method, similar to previously reported^34^,^35^,^36^,^37^,^38^. Black phosphorus crystal (smart elements) were used to exfoliate multilayers of black phosphorus flakes. The exfoliated samples were deposited on SiO_2_/Si substrate, with SiO_2_ thickness of 280 nm. Optical contrast was used to identify the relative thickness of the black phosphorus thin films. Flakes with dimensions larger than 5 μm were used for the spatial Raman mapping experiments. Samples were kept in ambient conditions and were monitored on a periodic manner.

### PMMA passivation

For PMMA coated samples, we spin coated PMMA immediately after exfoliation with different speeds for 1 minute in order to fully passivate the flake. After PMMA coating, some samples were optically measured in order to assess the degradation period. Other samples were measured before PMMA coating and were kept in the dark for a certain period of time until PMMA is removed. Optical measurements were carried out after PMMA removal.

### Raman spectroscopy characterization

confocal μ-Raman spectroscopy with automated stage (Renishaw) was used to characterize and perform the fast-scanning confocal Raman microscopy. A step size of 100 nm was used to ensure the measurements produce Raman maps with high confocality. Raman maps were produced using 100X objective lens using 532 nm laser as the excitation wavelength with low laser power and short exposure time.

## Additional Information

**How to cite this article:** Alsaffar, F. *et al*. Raman Sensitive Degradation and Etching Dynamics of Exfoliated Black Phosphorus. *Sci. Rep.* **7**, 44540; doi: 10.1038/srep44540 (2017).

**Publisher's note:** Springer Nature remains neutral with regard to jurisdictional claims in published maps and institutional affiliations.

## Supplementary Material

Supplementary Information
