# Supplementary material for: Raman Sensitive Degradation and Etching Dynamics of Exfoliated Black Phosphorus
Source: Sci Rep. 2017 Mar 20;7:44540. doi: 10.1038/srep44540 (PMC5357941; doi:10.1038/srep44540)
Supplement: Supplementary Information [file srep44540-s1.pdf]

Raman Sensitive Degradation and Etching Dynamics of Exfoliated Black Phosphorus Supplementary Document

Fadhel Alsaffar[1], Sarah Alodan[1], Abdullah Alrasheed[1], Abdulrahman Alhussain[1], Noura Alrubaiq[1], Ahmad Abbas[2,3,4], Moh. R. Amer[1,5]*

[1]Center of Excellence for Green Nanotechnologies,
Joint Centers of Excellence Program
King Abdulaziz City for Science and Technology
P.O Box 6086, Riyadh 11442, Saudi Arabia

[2]Department of Electrical Engineering
University of Southern California, Los Angeles, CA, 90089, USA

[3]Department of Electrical and Computer Engineering
University of Jeddah, 285 Dhahban 23881, Saudi Arabia

[4]King Abdulaziz University, Abdullah Sulayman Street, Jeddah 22254, Saudi Arabia

[5]Department of Electrical Engineering
420 Westwood Plaza, 5412 Boelter Hall
University of California, Los Angeles, Los Angeles, CA, 90095, USA

*Please send all correspondence to mamer@seas.ucla.edu, mamer@kacst.edu.sa

Table of contents:

1- Raman spectroscopy setup and Angle dependence of layered black phosphorus.
2- Optical and AFM measurements on the flake in figure 2.
   I. Additional Spatial Raman images of A_g^1, B_{2g}, and A_g^2 Raman modes at different time intervals for the sample in figure 2.
   II. AFM measurements of the sample in figure 2.
3- Degradation Study of few layers black phosphorus.
   I. Spatial Raman Intensity Mapping:
   II. Raman characteristics at a specific site on the flake.
4- Additional Samples showing the modulation in Raman intensity with degradation.
   I. Other flakes that showed the intensity change.
   II. Monitoring the degradation of thick flake.
      i. Raman intensity maps at different time intervals
      ii. Raman intensity at a specific point on the flake.
      iii. AFM measurements on *t*=23 days.
5- PMMA coated samples.
6- Raman intensity enhancement model:
   I. Model details.
   II. Thickness and etching rate estimation.

7- Effect of liquid interfaces on the Raman intensity modulation.

1- Raman spectroscopy setup and Angle dependence of layered black phosphorus.

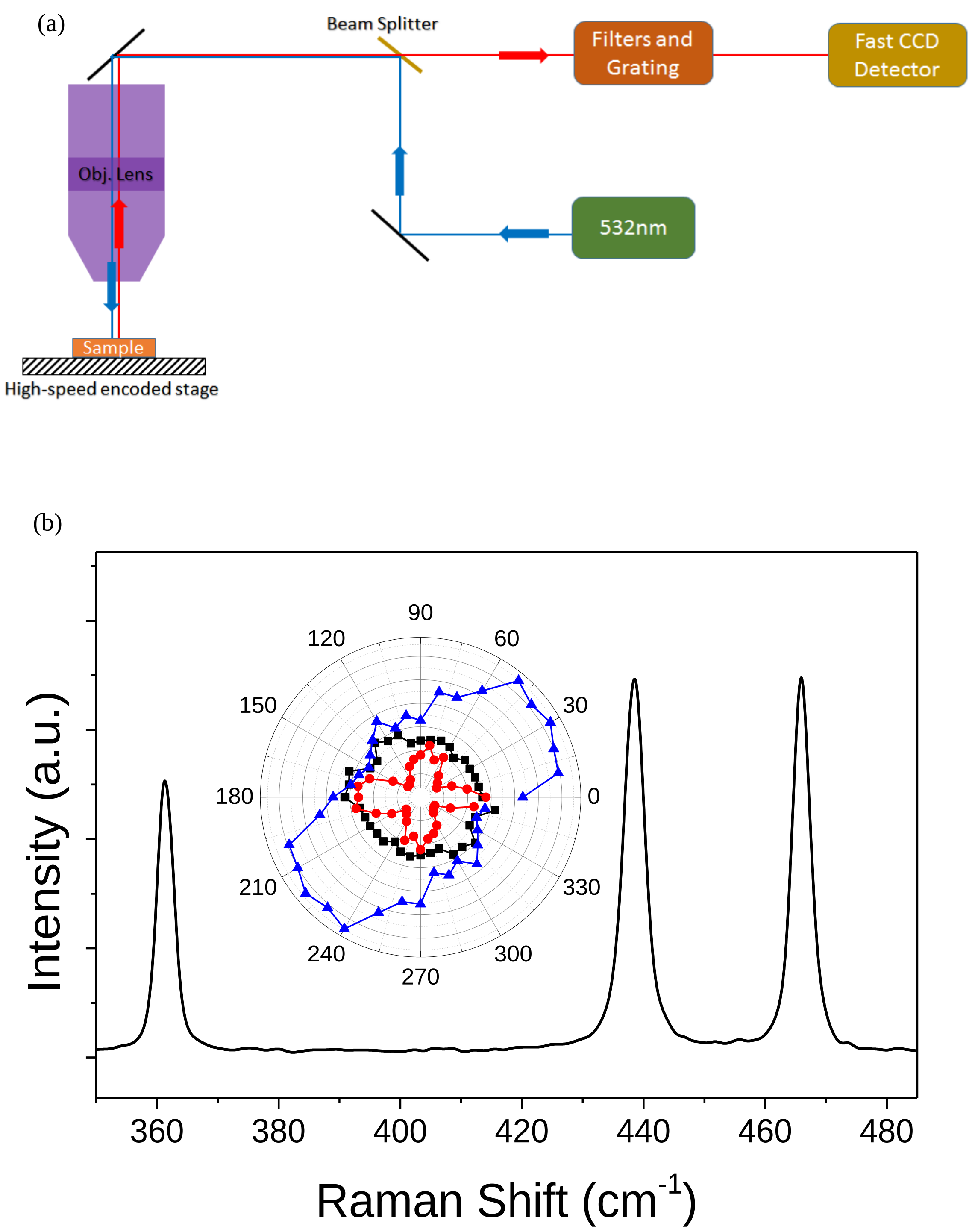

Figure S1. (a) Setup schematics of fast-scanning confocal Raman spectroscopy of layered black phosphorus. (b) Typical Raman spectrum of layered black phosphorus. The inset shows the angle dependence of A_g^1 (black), B_{2g} (red), and A_g^2 (blue) modes.

2- Optical and AFM measurements on the flake in figure 2.

a. Additional Spatial Raman images of A_g^1, B_{2g}, and A_g^2 Raman modes at different time intervals for the sample in figure 2:

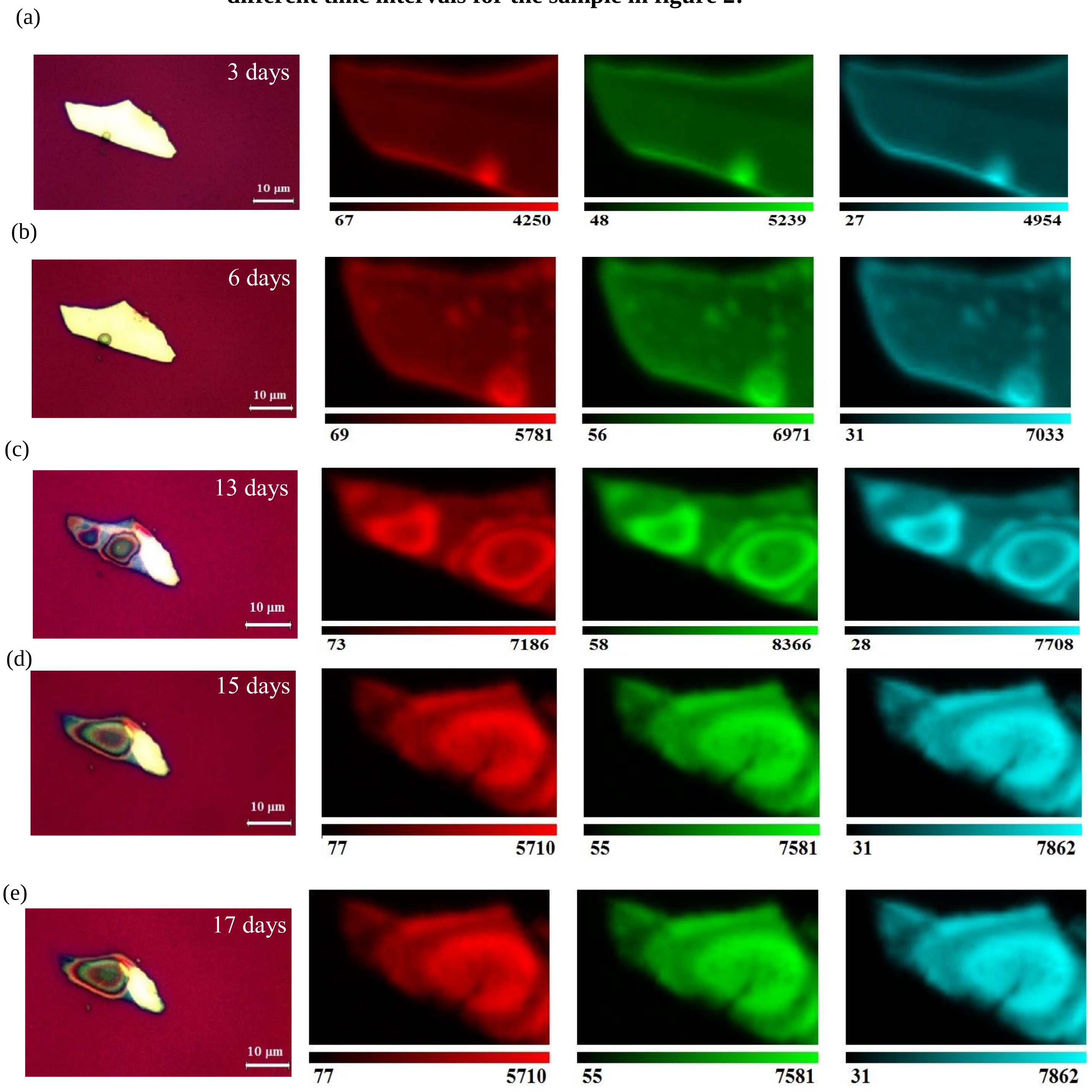

Figure S2. (a) Optical image and the corresponding Raman intensity maps of A_g^1 (cyan color), B_{2g} (red color), and A_g^2 (green color) vibrational peaks for the flake in figure 1 at (a) t= 3 days, (b) t = 6 days, (c) t = 13 days, (d) t = 15 days, and (e) t = 17 days.

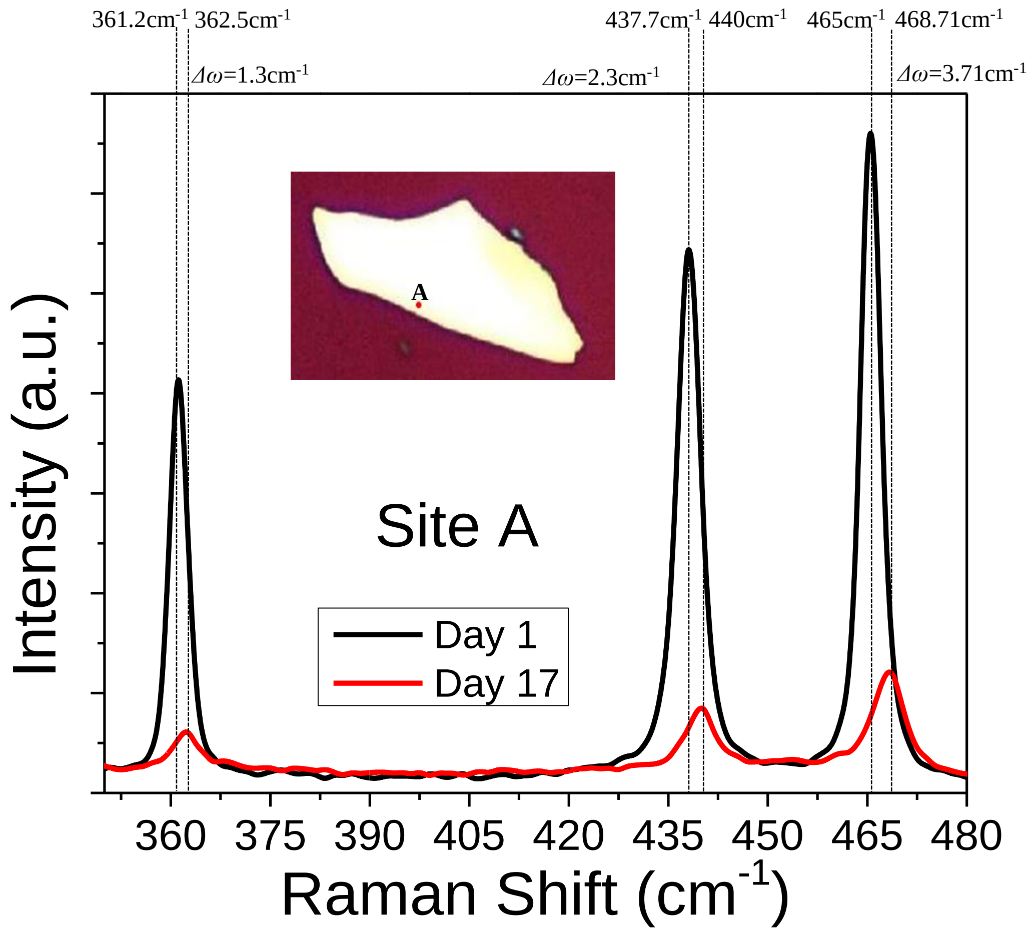

Figure S3. Raman spectra showing the Raman shift of each Raman mode for the thin film flake in figure 2. The spectra are taken at t=1days and t=17days.

b. AFM measurements of the sample in figure 2.

We carried out AFM measurements of the black phosphorus thin film flake in figure 2 to determine the initial thickness of the flake. We scanned the edge of the flake as shown in figure S2. In this image, the flat part is the non-degraded part, while the lower part that exhibit high intensity shows the bubble formation that was shown previously by Island *et al.*, where the volume of the black phosphorus flake has increased significantly after few days. The estimated flake thickness that was used in the intensity enhancement model is d = 35nm ± 5nm.

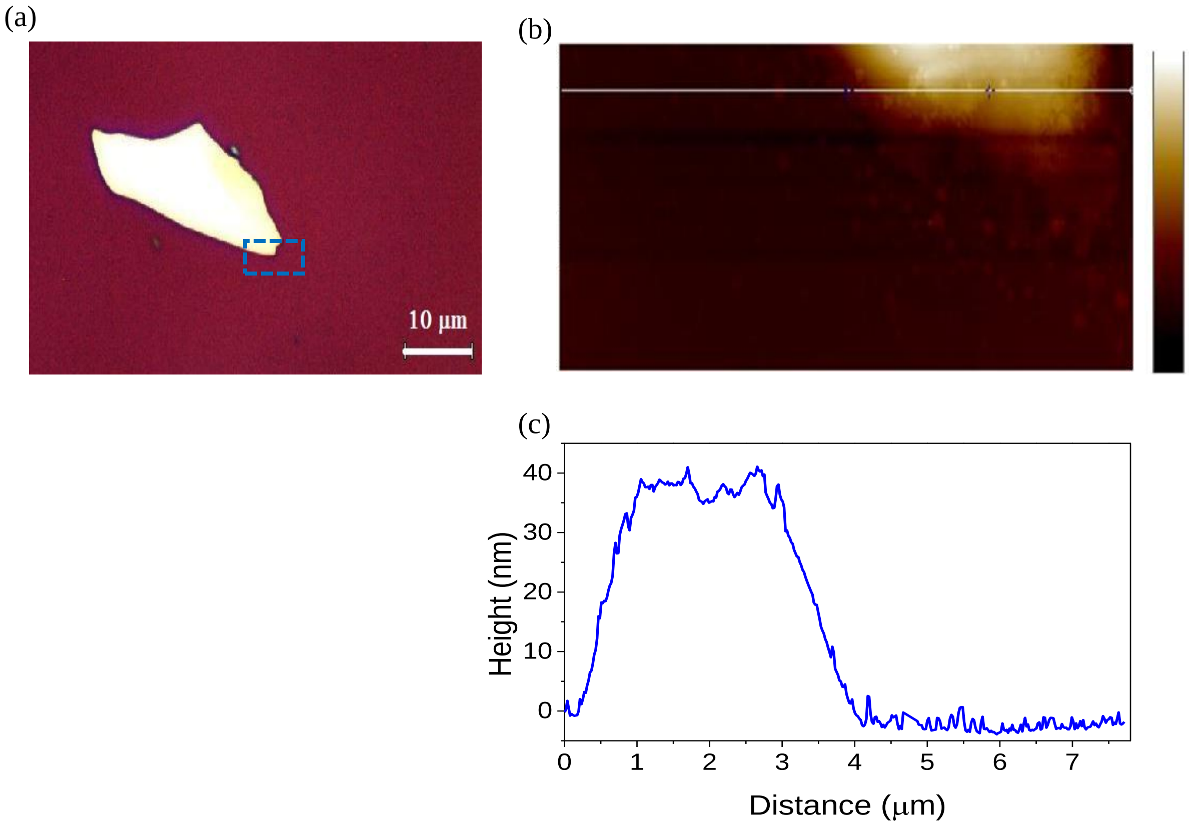

Figure S4. (a) Optical image and the black phosphorus thin film flake. The highlighted region is the AFM scanning region. (b) AFM image of the flake in (a) after few days of exfoliation. The flat part shows the original flake thickness. (c) The height profile along the horizontal white line in the AFM image in (b).

3- Degradation Study of few layers black phosphorus.

b. Spatial Raman Intensity Mapping:

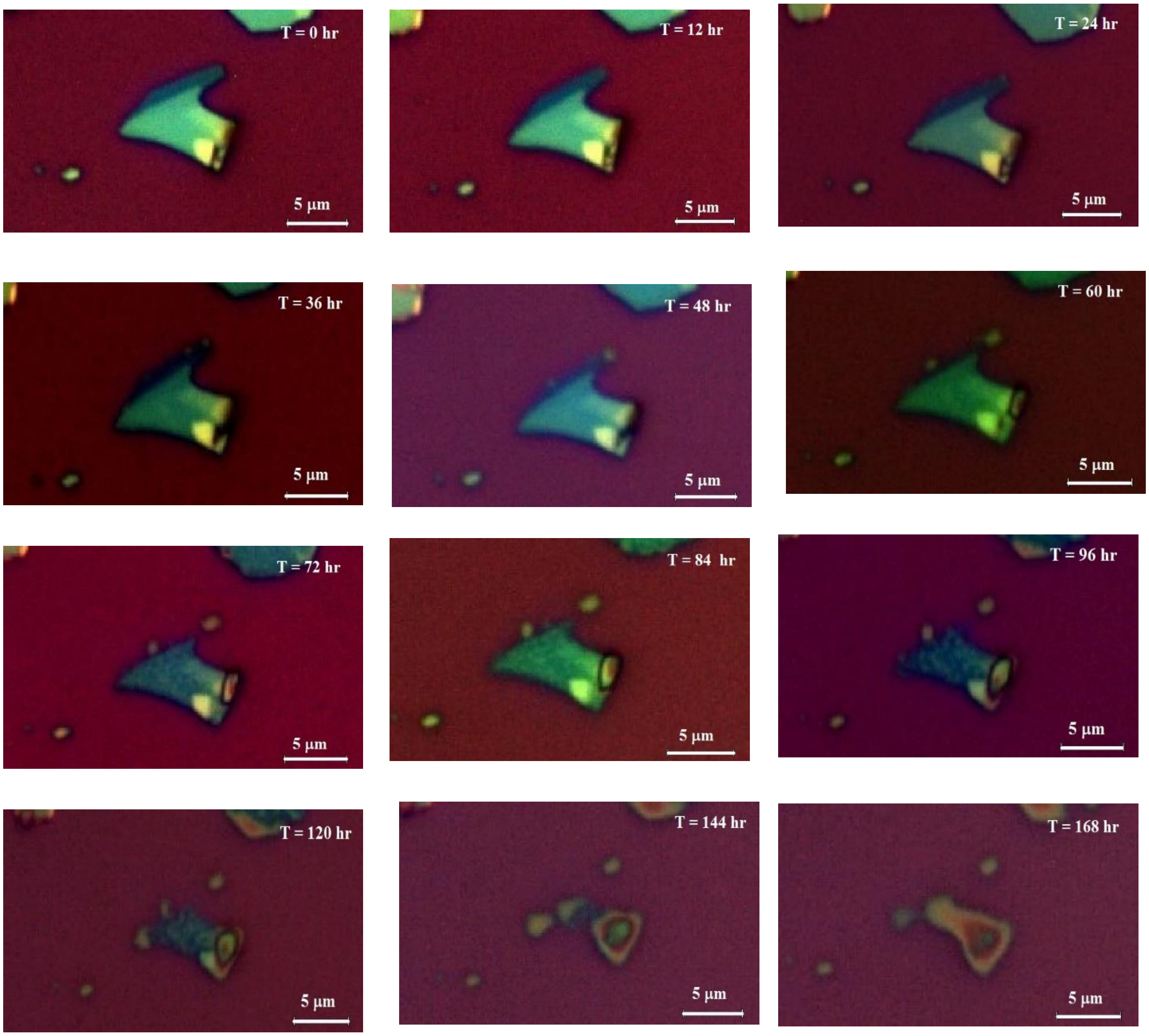

Figure S5. (a) Optical images of few layers black phosphorus showing the degradation at different times. As shown, few layers black phosphorus reacts with ambient environment in hours.

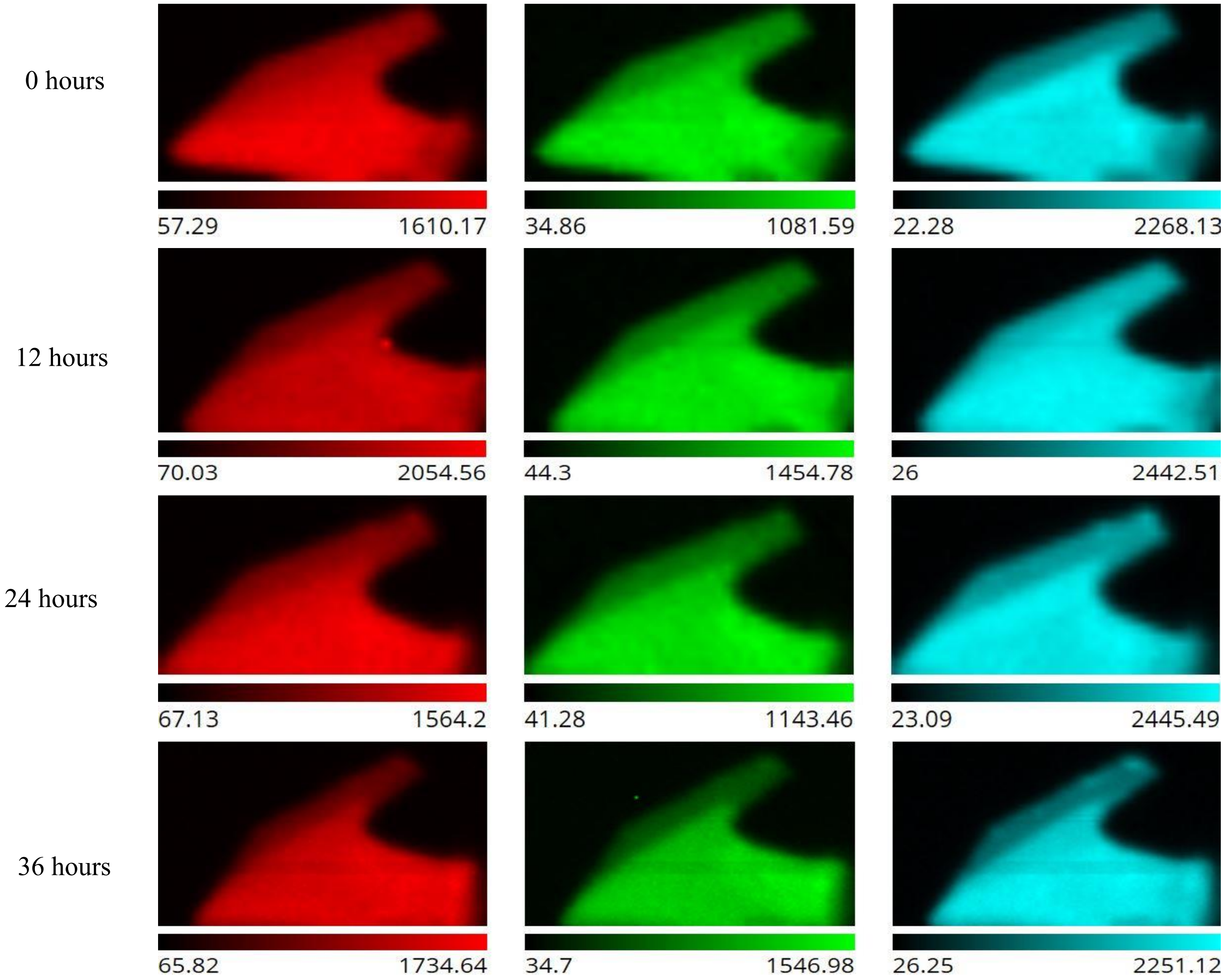

0 hours
57.29
1610.17
34.86
1081.59
22.28
2268.13
12 hours
70.03
2054.56
44.3
1454.78
26
2442.51
24 hours
67.13
1564.2
41.28
1143.46
23.09
2445.49
36 hours
65.82
1734.64
34.7
1546.98
26.25
2251.12

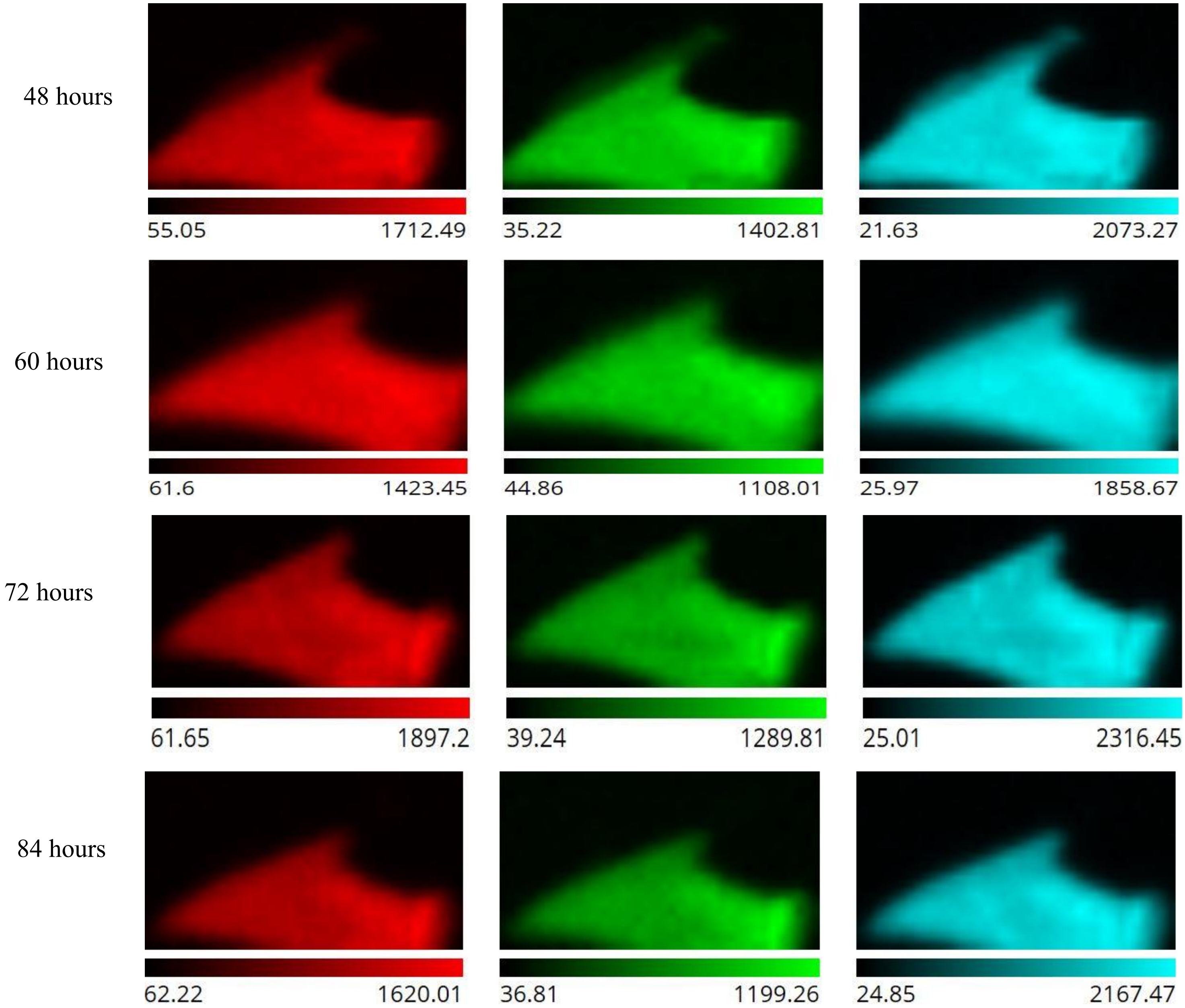
48 hours
55.05
1712.49
35.22
1402.81
21.63
2073.27
60 hours
61.6
1423.45
44.86
1108.01
25.97
1858.67
72 hours
61.65
1897.2
39.24
1289.81
25.01
2316.45
84 hours
62.22
1620.01
36.81
1199.26
24.85
2167.47

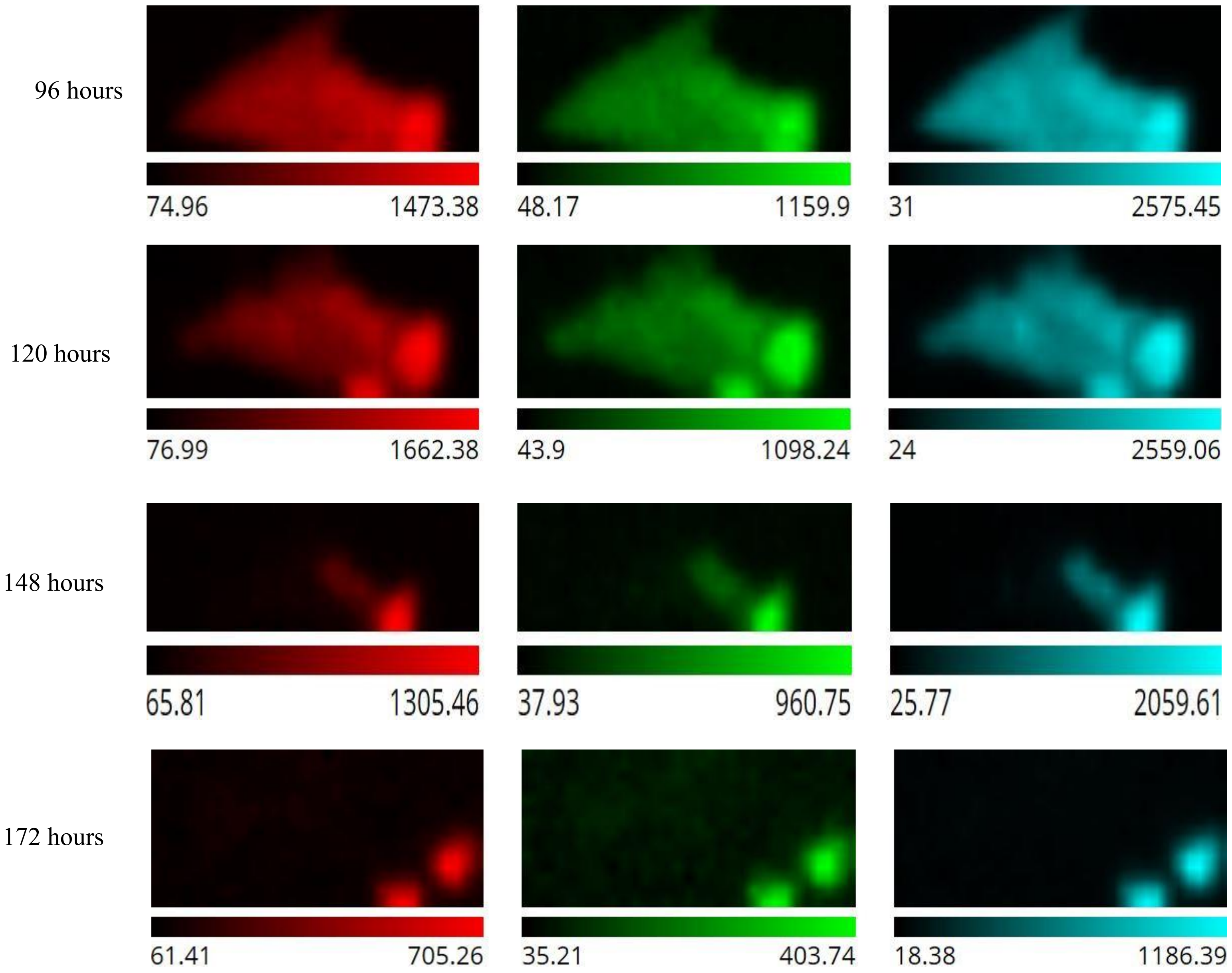

Figure S6. Raman Intensity maps of A_g^1 (red color), B_{2g} (green color), and A_g^2 (cyan color) vibrational peaks of the few layers flake in figure S3 at different time intervals.

c. Raman characteristics at a specific site on the flake.

The Raman characteristics of a specific site on few layers black phosphorus flake are shown in figure S7 for A_g^1, B_{2g}, and A_g^2 Raman modes. The Intensity of each mode shows a decrease, analogous to previous reports [1]. Also, we observe slight Raman upshift before the flake completely degrades. This Raman upshift reflects layers lowering, which can be interpreted as layers etching [1, 2].

(a)

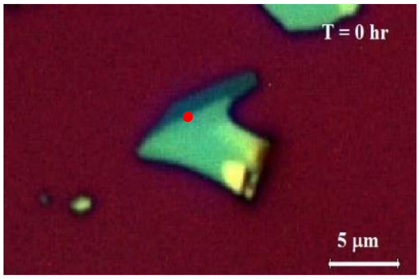

(b)

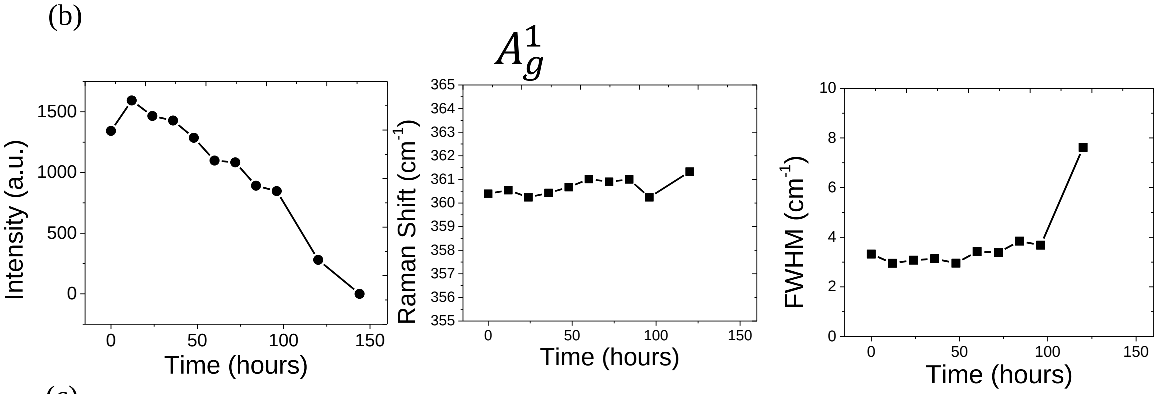

(c)

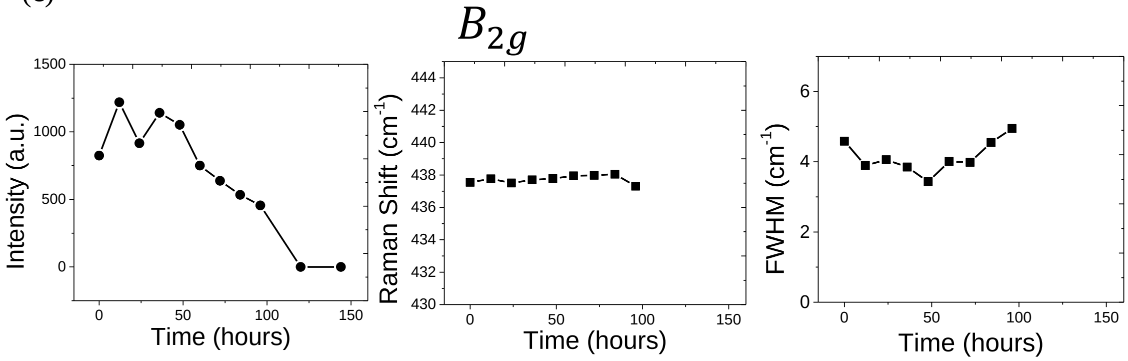

(d)

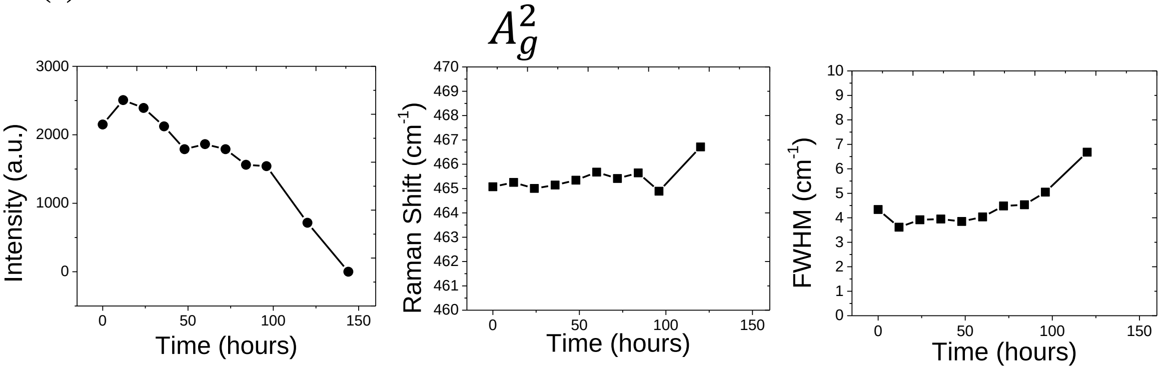

4- Additional Samples showing the modulation in Raman intensity with degradation.

a. Other flakes that showed the intensity change.

Figure S7. (a) optical image showing the site of interest (red dot). The Raman intensity, Raman shift, and FWHM of the highlighted site in A is shown for (b) A_g^1, (c) B_{2g}, and (d) A_g^2.

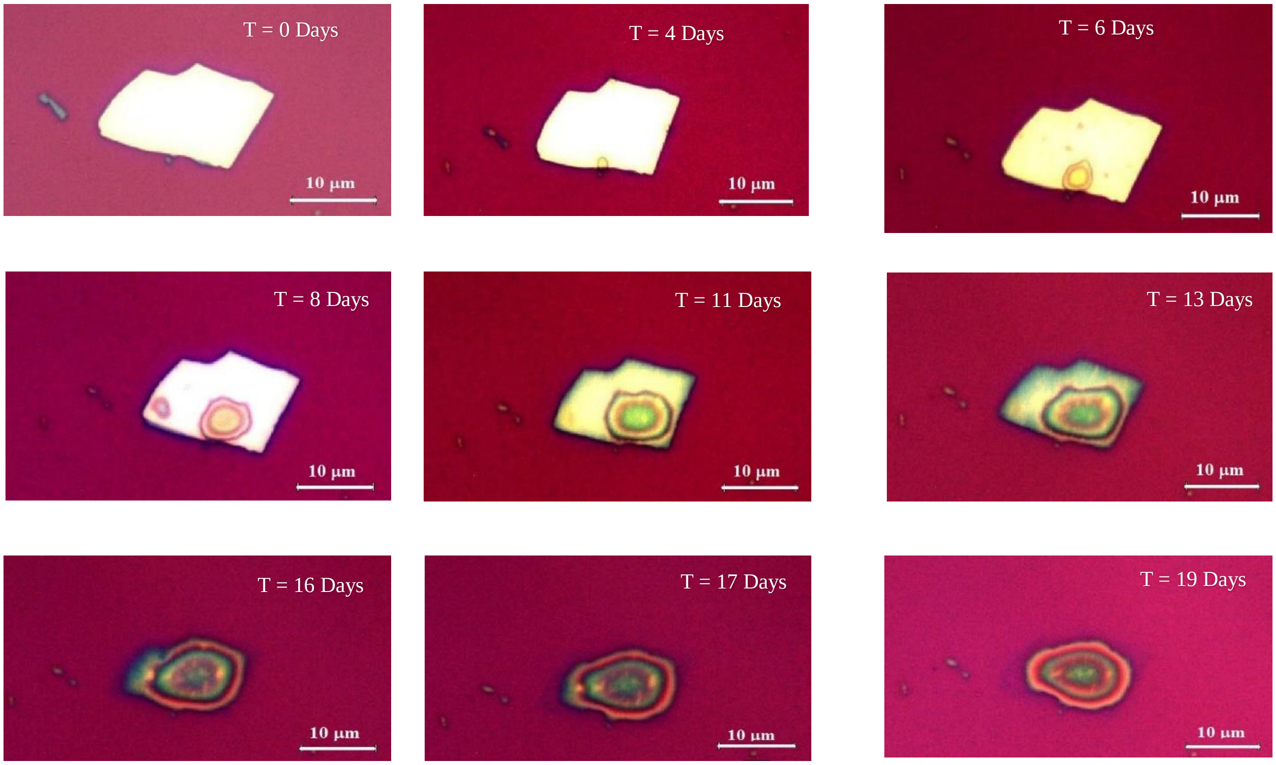

Figure S8. Optical images at different time intervals for multilayer black phosphorus showing edge degradation as the dominant mechanism.

0 Days

4 Days

6 Days

8 Days

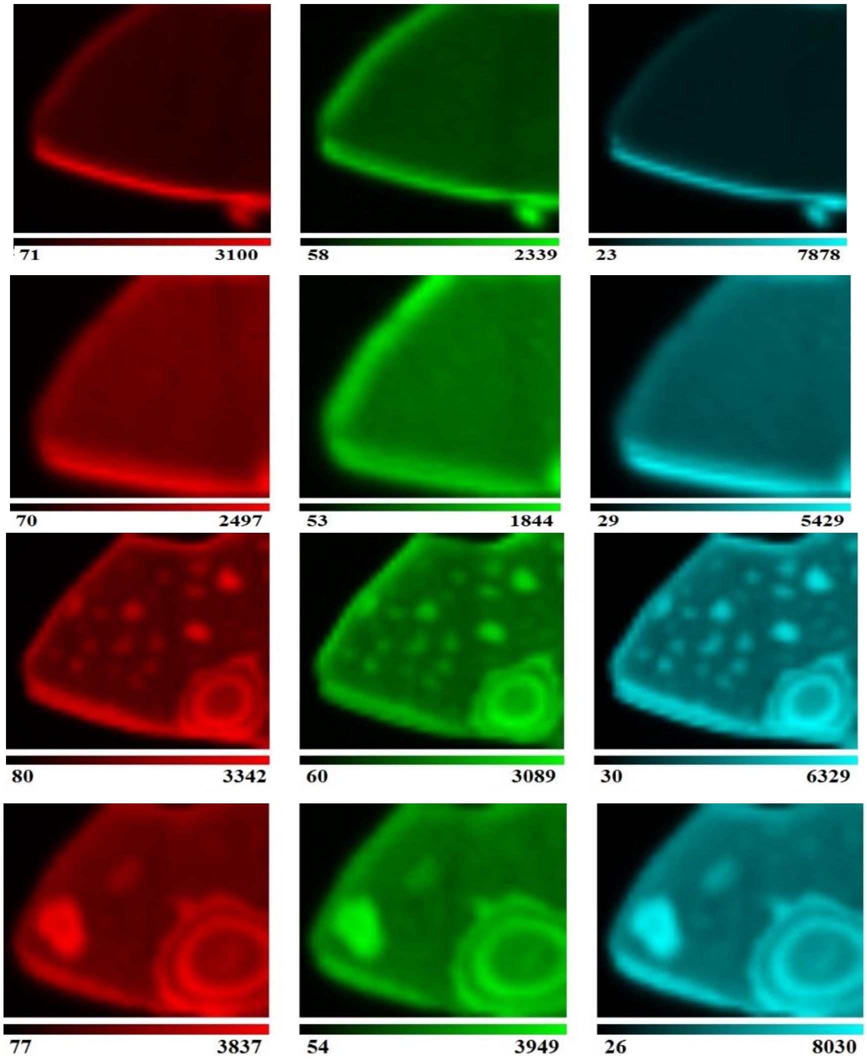

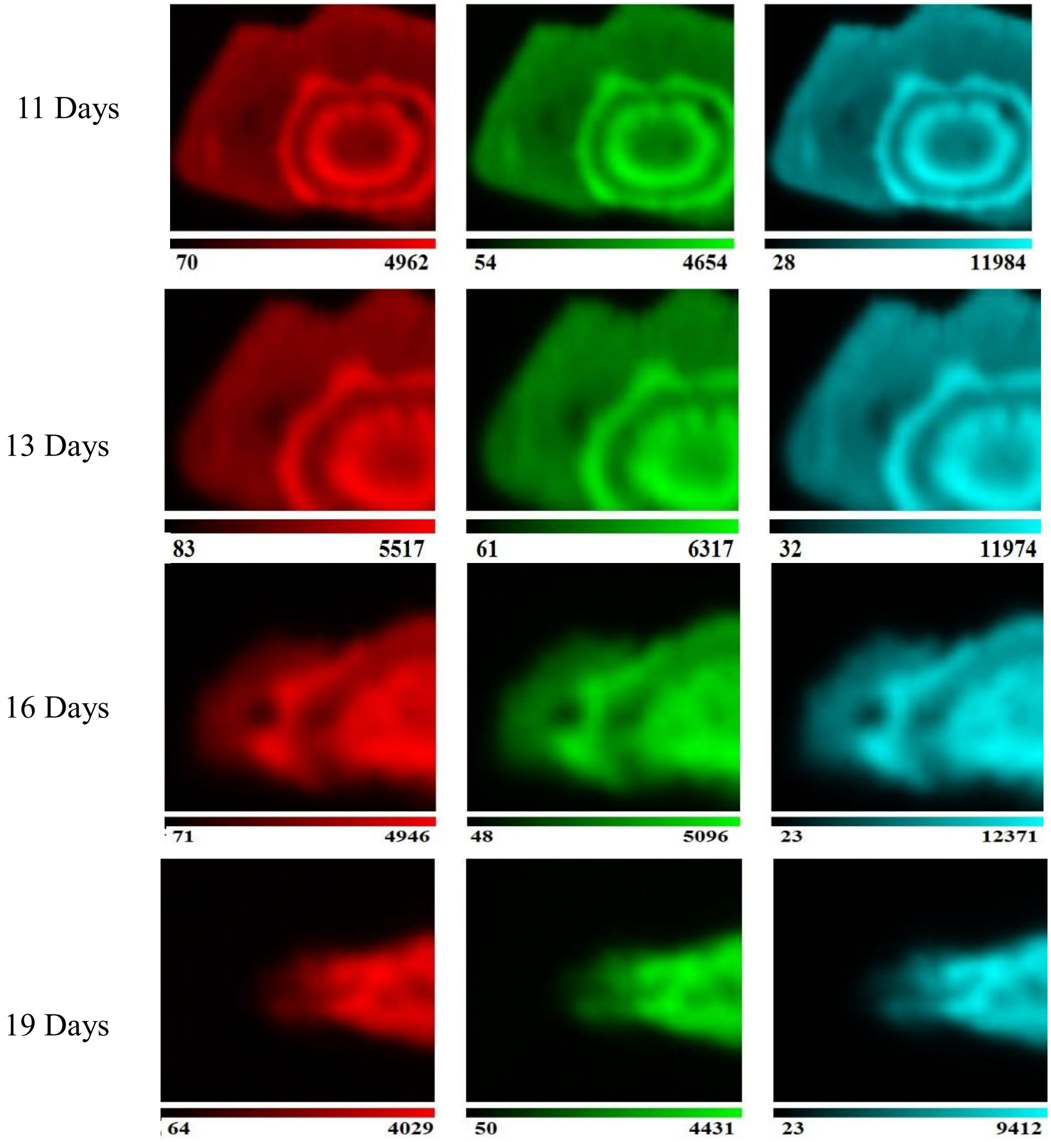

Figure S9. Raman Intensity maps of A_g^1 (red color), B_{2g} (green color), and A_g^2 (cyan color) vibrational peaks of the flake in figure S5 at different time intervals.

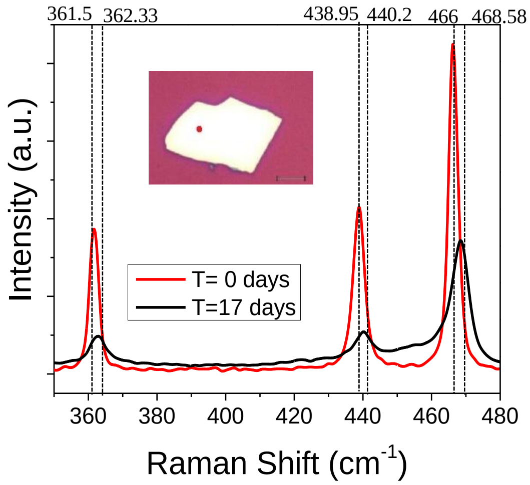

Figure S10. Raman spectra showing the Raman shift of each Raman mode for the thin film flake above. The spectra are shown for the highlighted site in the inset.

.

b- Monitoring the degradation of thick flake:

I. Raman intensity maps at different time intervals

We exfoliated a thick black phosphorus flake as shown in figure S7. The flake intial thickness is is estimated to be as large as ~200nm. The sheet shows edge degradation as the first sign of degradation in the optical image for *T*=3days. Subsequently, surface degradation starts to show on the surface of the sheet. The sheet completely degrades after 57 days. We monitored the Raman maps of each of A_g^1, B_{2g}, and A_g^2 Raman modes at different time intervals in order to assess the mechanism of the degradation (figure S8).

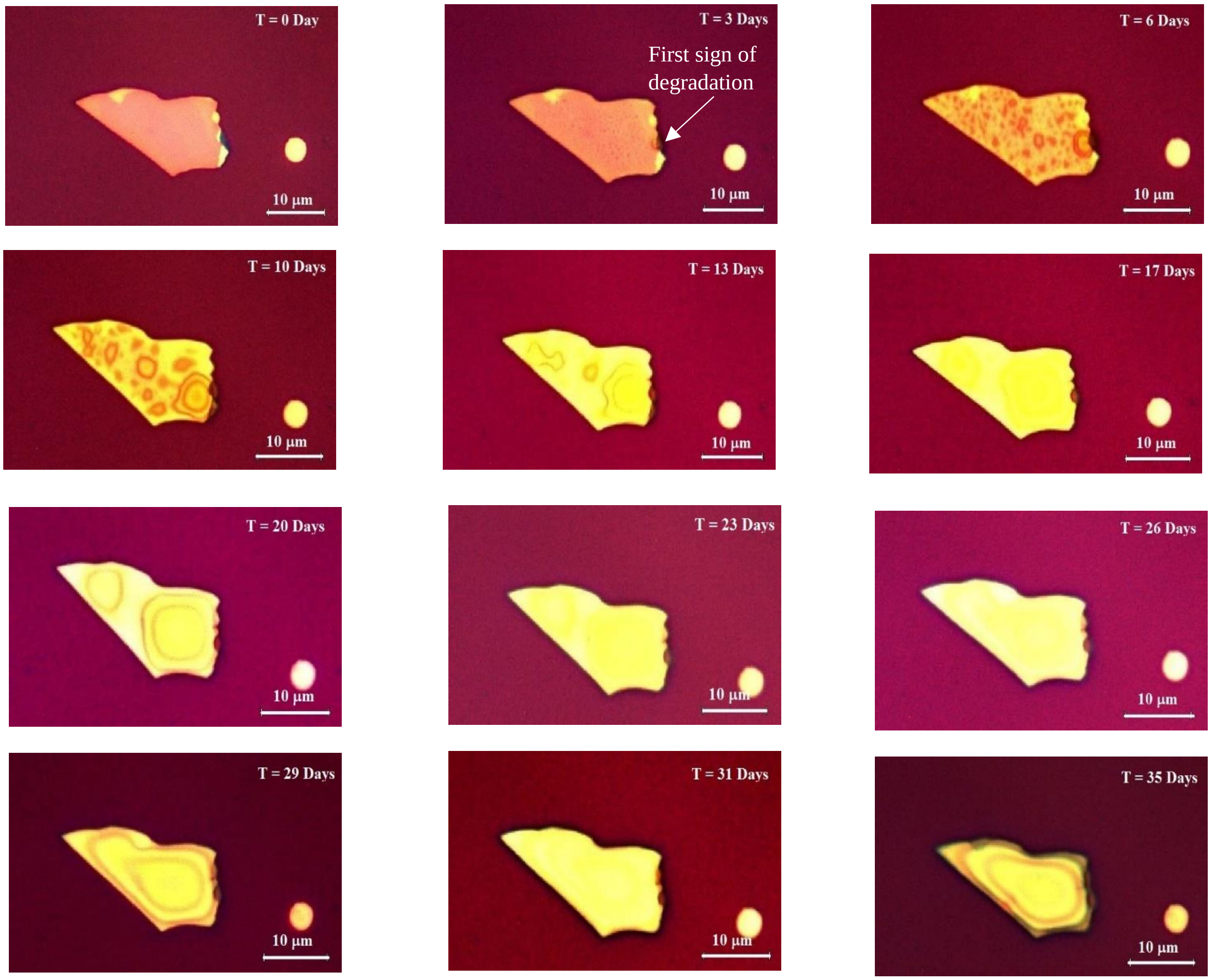

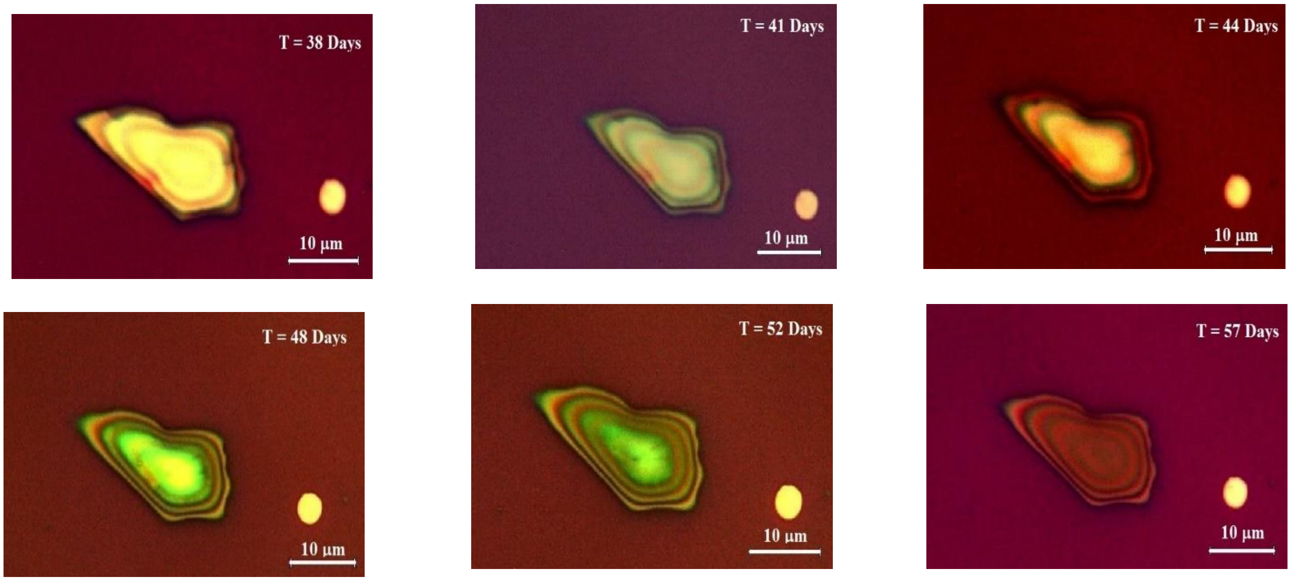

Figure S11. Optical images at different time intervals for thick black phosphorus. The flake degrades completely by the end of 57 days.

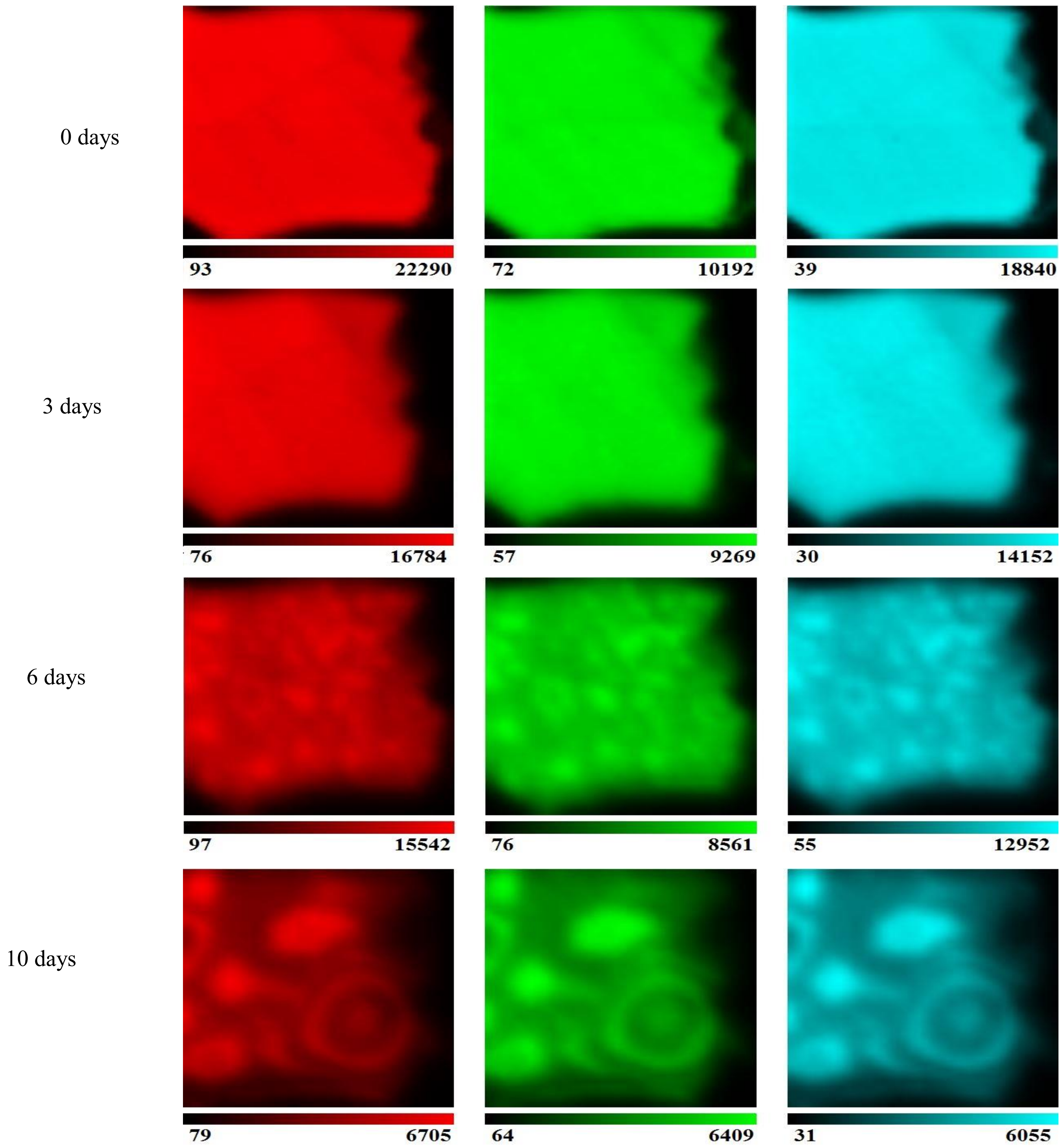

0 days
93
22290
72
10192
39
18840
3 days
76
16784
57
9269
30
14152
6 days
97
15542
76
8561
55
12952
10 days
79
6705
64
6409
31
6055

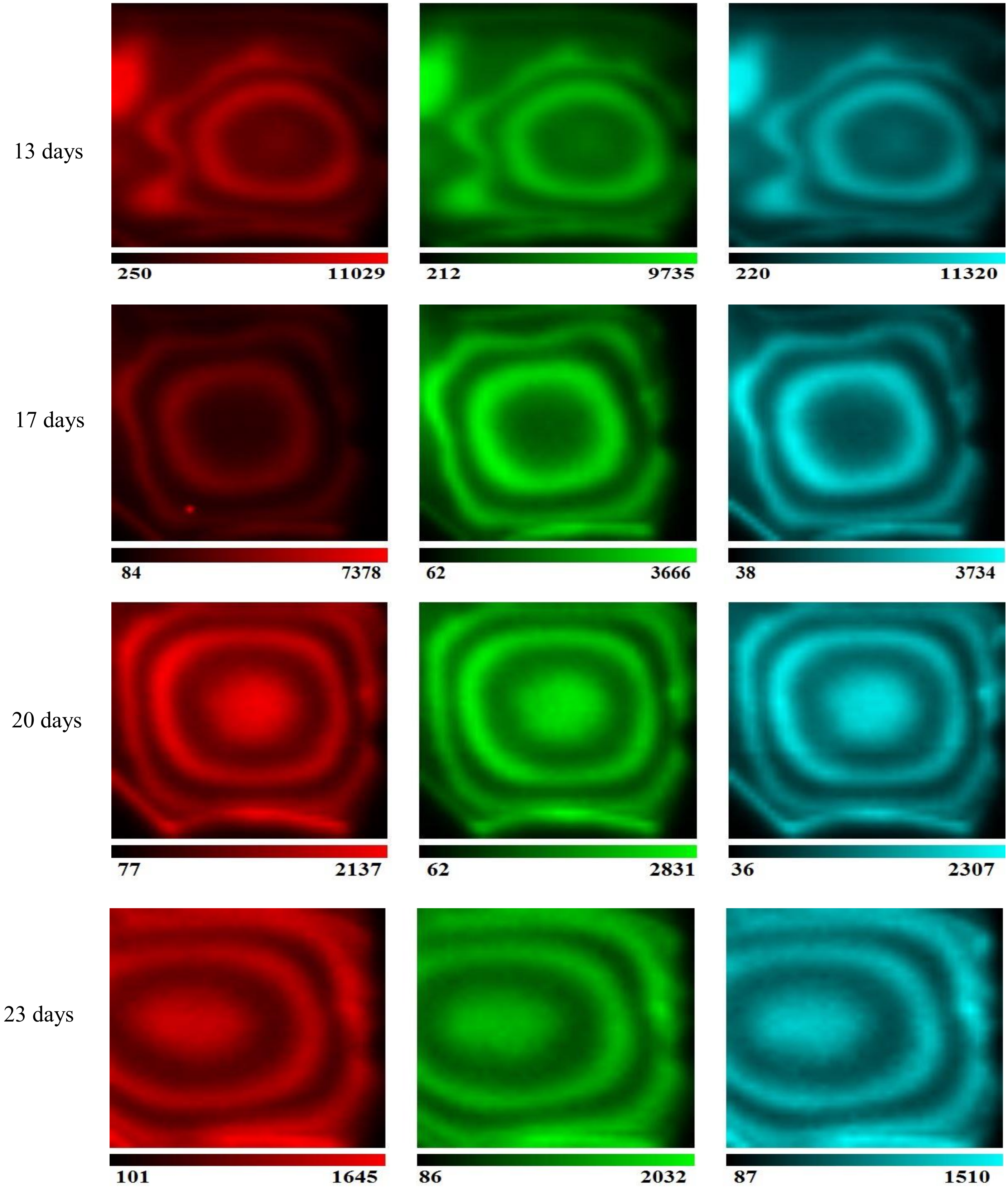

13 days
250
11029
212
9735
220
11320
17 days
84
7378
62
3666
38
3734
20 days
77
2137
62
2831
36
2307
23 days
101
1645
86
2032
87
1510

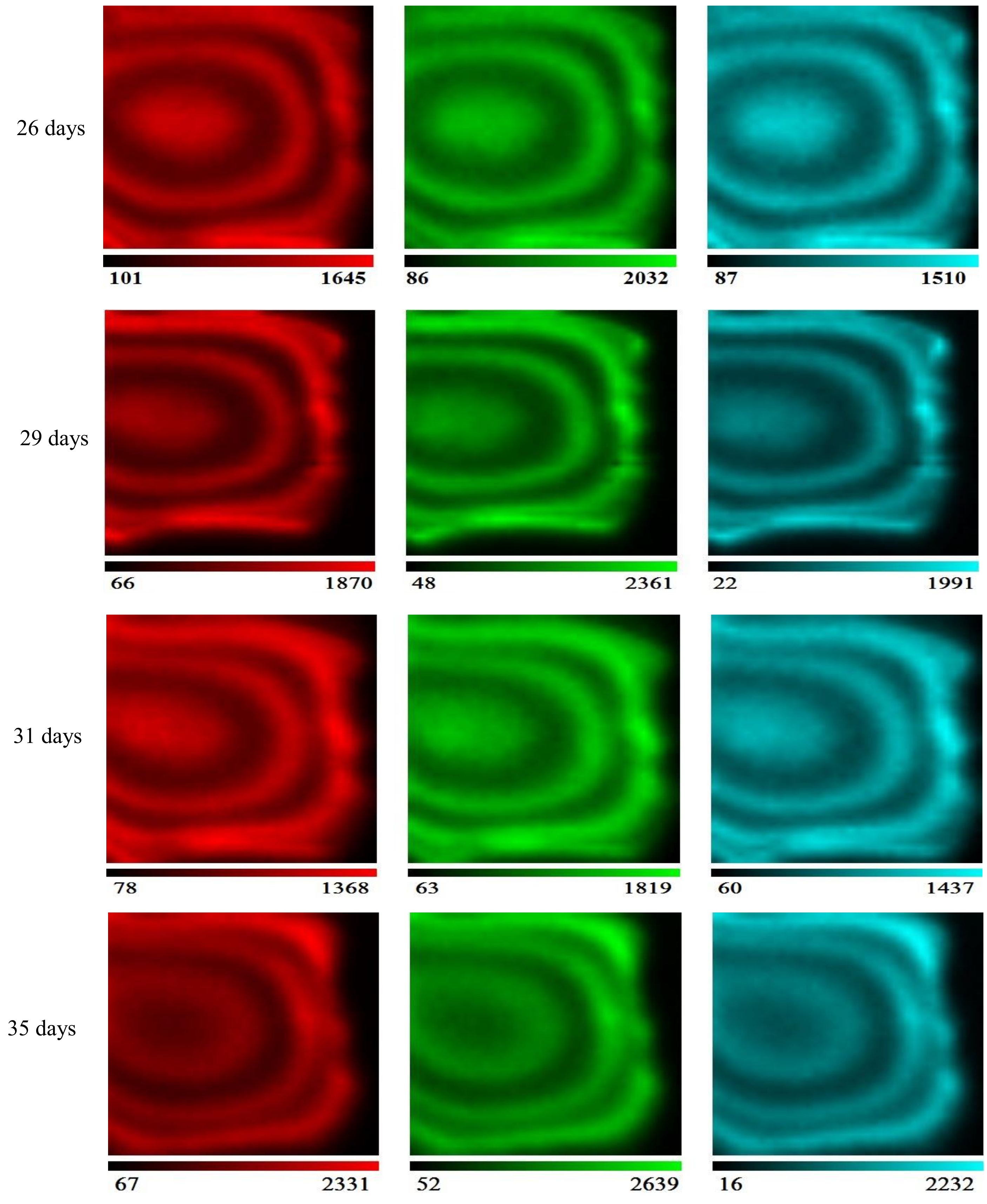
26 days
101
1645
86
2032
87
1510
29 days
66
1870
48
2361
22
1991
31 days
78
1368
63
1819
60
1437
35 days
67
2331
52
2639
16
2232

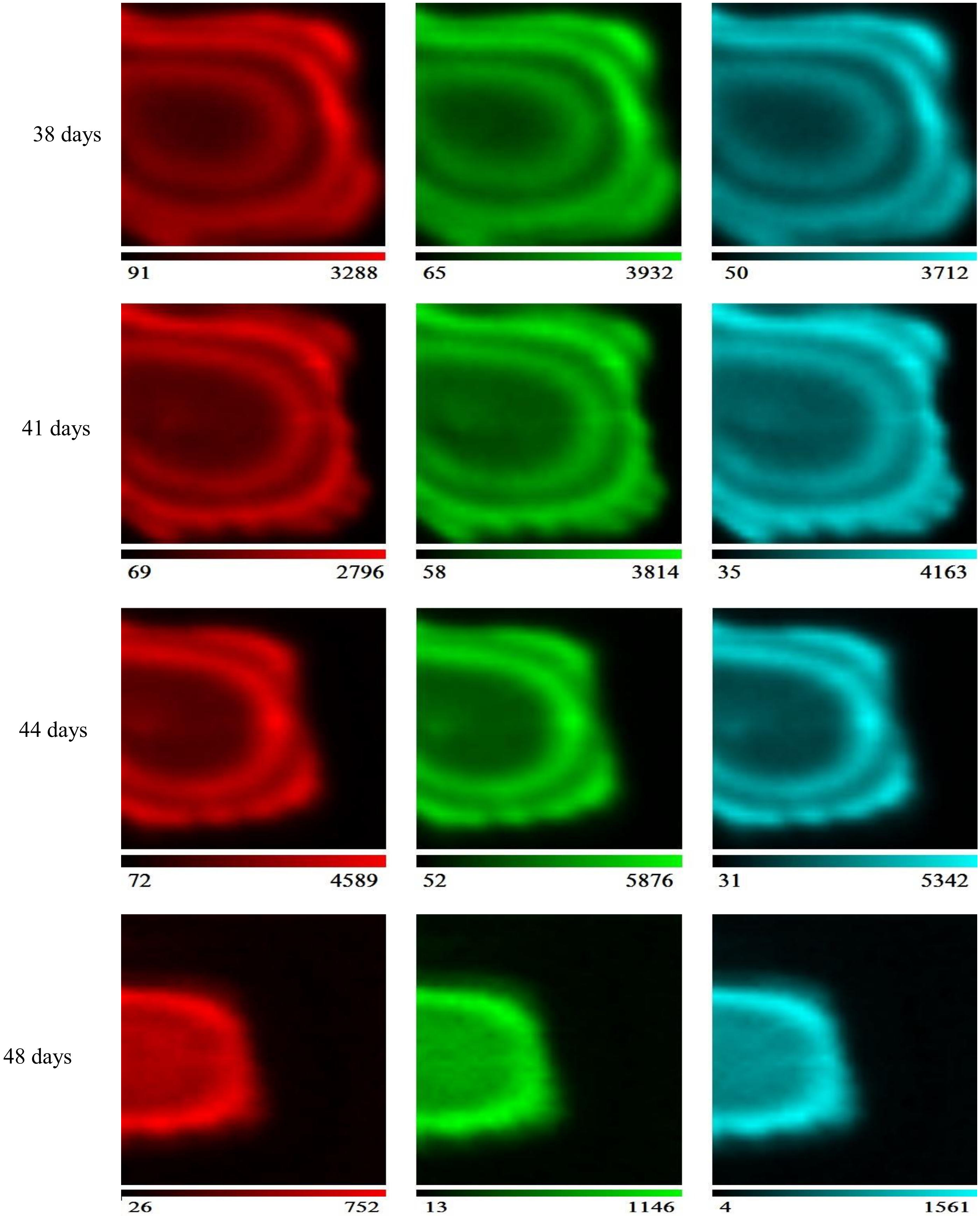
38 days
91
3288
65
3932
50
3712
41 days
69
2796
58
3814
35
4163
44 days
72
4589
52
5876
31
5342
48 days
26
752
13
1146
4
1561

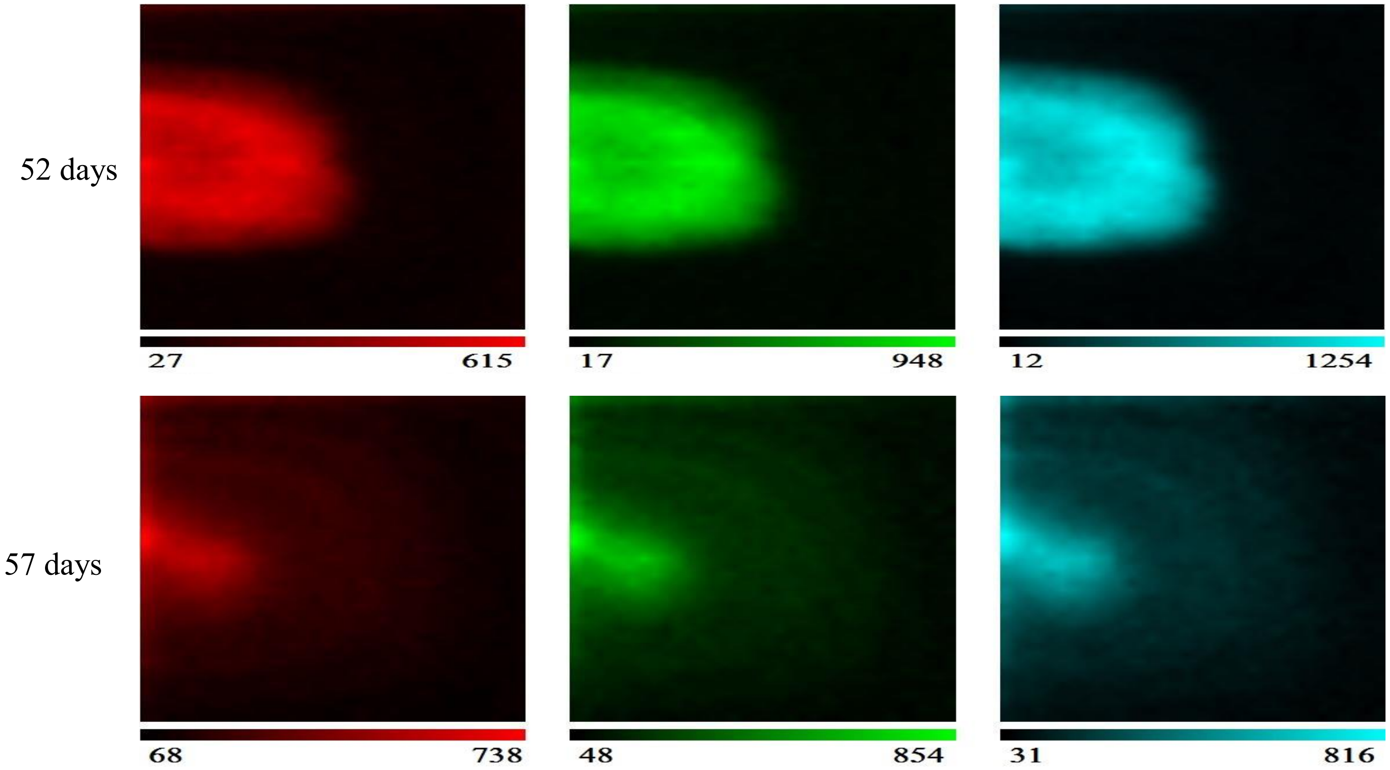

Figure S12. Raman Intensity maps of A_g^1 (red color), B_{2g} (green color), and $^{2}_{g}$ (cyan color) vibrational peaks of the flake in figure S7 at different time intervals.

II. Raman intensity at a specific point on the flake.

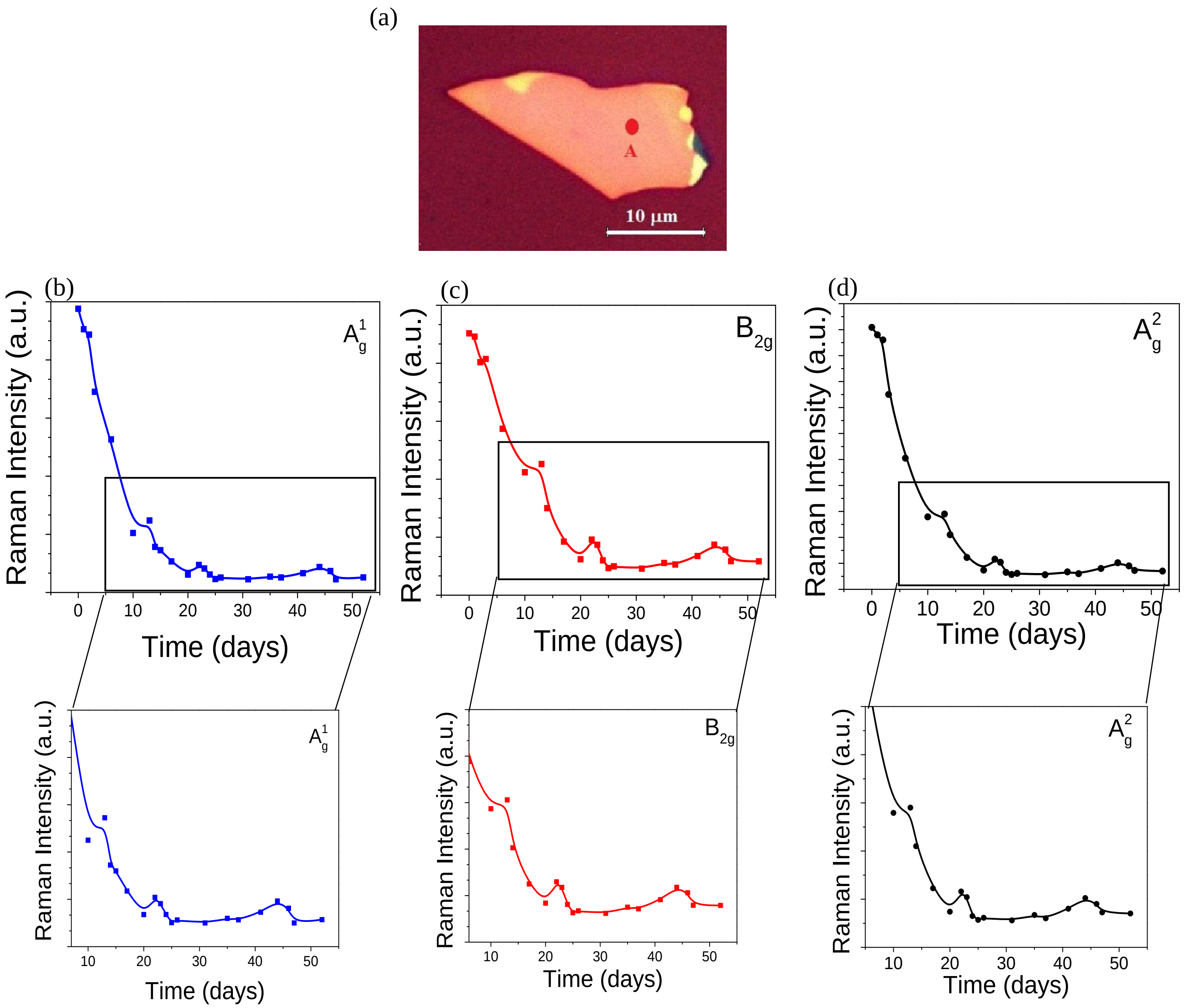

Figure S13. Raman intensity vs. time for (b) A_g^1, (c) B_{2g}, and (d) A_g^2 on a specific site on the flake as (a) highlighted in the optical image. Notice the intensity profile exhibit oscillatory behavior, analogous to the non-normalized intensity enhancement plot in figure S17a.

a. AFM measurements on *t*=23 days.

In order to validate the intensity enhancement model due to interference, AFM measurements have been carried out on a thick flake at a specific time interval in order to compare the thickness after degradation with the intensity enhancement model, Our AFM measurements are in good agreement with our measured Raman intensity modulation, further cooperating our assessment on the intensity enhancement due to optical interference. Below is the AFM image along with the line profile showing the thickness of black phosphorus for *t*=23 days.

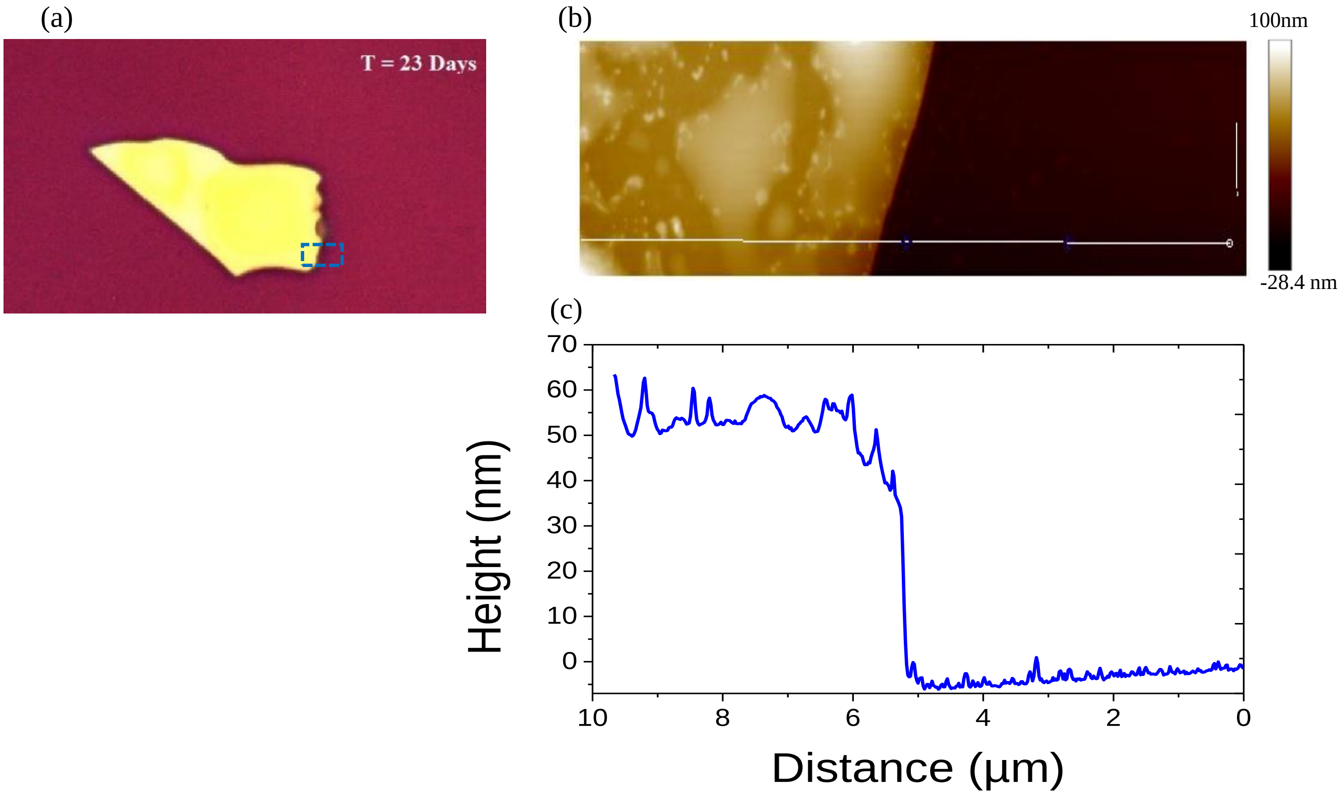

Figure S14. (a) Optical image and the black phosphorus thin film flake. The highlighted region is the AFM scanning region. (b) AFM image of the flake in (a) after *t*=23 days of exfoliation. (c) The height profile along the horizontal white line in the AFM image in (b).

5- PMMA coated few layers black phosphorus:

In order to assess the degradation rate for PMMA coated few layers black phosphorus, we monitored the flake degradation

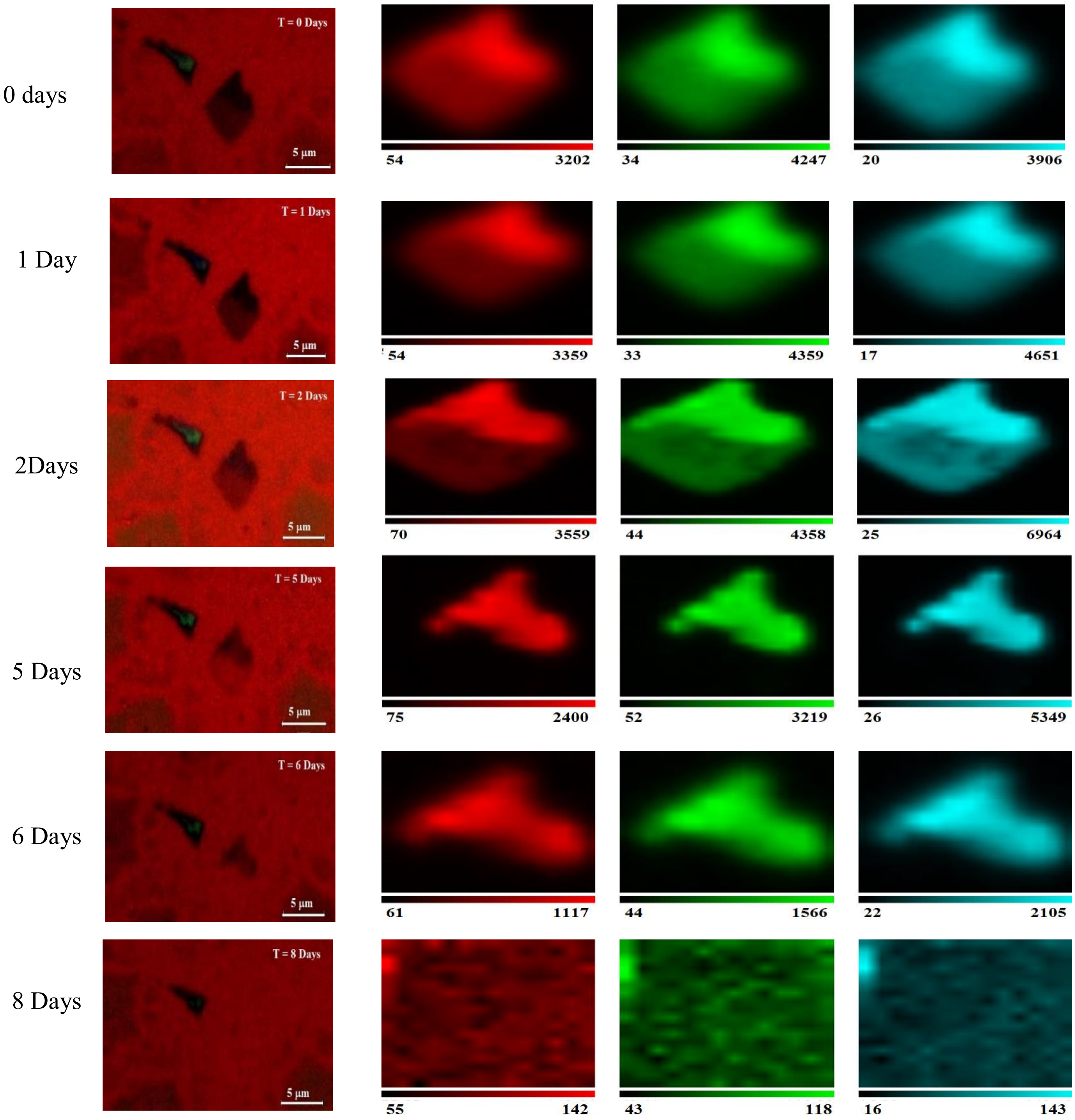

Figure S15. Optical images and Raman Intensity maps of A_g^1 (red color), B_{2g} (green color), and A_g^2 (cyan color) vibrational peaks for a few layers flake. The first signs of degradation occur on the second day.

6- Raman intensity enhancement model:

a. Model details:

In our calculations, there are two major processes take place that contribute to this Raman intensity modulation. The first process is the absorption of the incident laser on the black phosphorus thin film as illustrated in figure 3. The final net absorption is governed by:

$$F_{ab} = t_1 \frac{[1+r_2 r_3 e^{-2iB_2}]e^{-iB_X}+[r_2+r_3 e^{-2iB_2}]e^{-i(2B_1-B_X)}}{1+r_2 r_3 e^{-2iB_2}+(r_2+r_3 e^{-2iB_2})r_1 e^{-2iB_1}},$$

where $t_1 = \frac{2n_0}{(n_1+n_0)}$, $r_1 = \frac{n_0-n_1}{(n_0+n_1)}$, $r_2 = \frac{n_1-n_2}{(n_1+n_2)}$, and $r_3 = \frac{n_2-n_3}{(n_2+n_3)}$ are the transmission coefficients. n_0, n_1, n_2 and n_3 are the refractive indices for air, BP sheet, SiO_2, and Si, respectively. These values are found in ref [3, 4]. d_1 and d_2 are the thickness of as exfoliated BP sheet and SiO_2. X is the depth in the BP sheet that the laser is being absorbed and scattered. The coefficients B_X, B_2,and B_2 are given by $B_X = \frac{2\pi X n_1}{\lambda_{ex}}$, $B_1 = \frac{2\pi d_1 n_1}{\lambda_{ex}}$, $B_2 = \frac{2\pi d_2 n_2}{\lambda_{ex}}$, where λ_{ex} is the excitation wavelength of the laser (532nm in our case).

The second process is the Raman scattering interference, which occurs inside the BP sheet, as schematically shown in figure S10b. The scattering term (F_{sc}) can be calculated according to:

$$F_{sc} = t_2 \frac{[1+r_2 r_3 e^{-2iB_2}]e^{-iB_X}+[r_2+r_3 e^{-2iB_2}]e^{-i(2B_1-B_X)}}{1+r_2 r_3 e^{-2iB_2}+(r_2+r_3 e^{-2iB_2})r_1 e^{-2iB_1}},$$

where $t_2 = \frac{2n_1}{(n_1+n_0)}$, and for the scattering terms are given by $B_x = \frac{2\pi X n_1}{\lambda_{sc}}$, $B_1 = \frac{2\pi d_1 n_1}{\lambda_{sc}}$, $B_2 = \frac{2\pi d_2 n_2}{\lambda_{sc}}$, where λ_{sc} is the desired Raman mode wavelength.

The net enhancement factor after taking the absorption and the Raman multiple scattered light into account is given by [5]:

$$F = N \int_0^{d_1} |F_{ab}\ F_{sc}|^2\ dx$$

Where N is the normalization factor and it is introduced in the equation to remove the effects of the substrate (SiO_2 and Si). Figure S17 plots the intensity enhancement factor with and without the normalization factor. In this figure, the intensity enhancement for the zigzag direction shows significant increase compared to the armchair direction.

Notice the non-normalized intensity enhancement factor exhibit similar profile to the measured Raman intensity for each Raman mode in figure S17a.

(a)

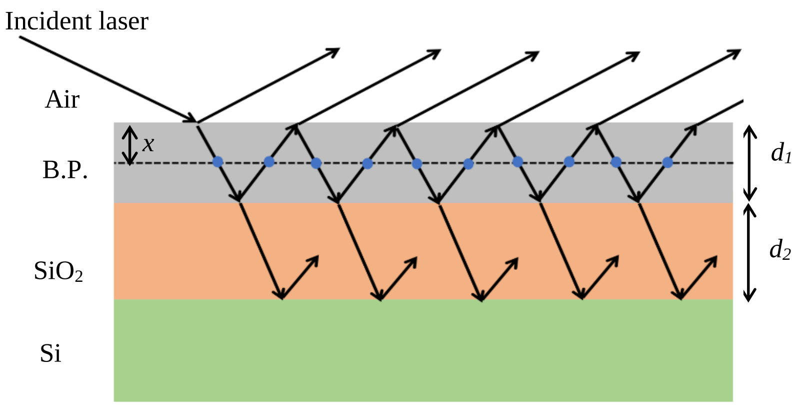

(b)

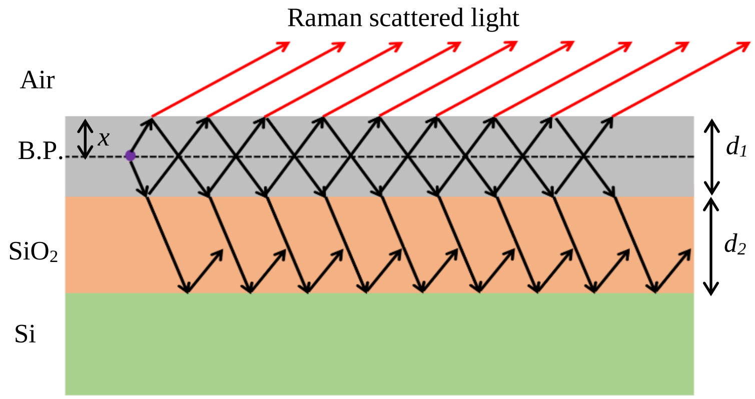

Figure S16. Schematic diagrams illustrating the multiple scattering processes in black phosphorus sheet on SiO2/Si substrates for (a) absorption, (b) Raman scattered light.

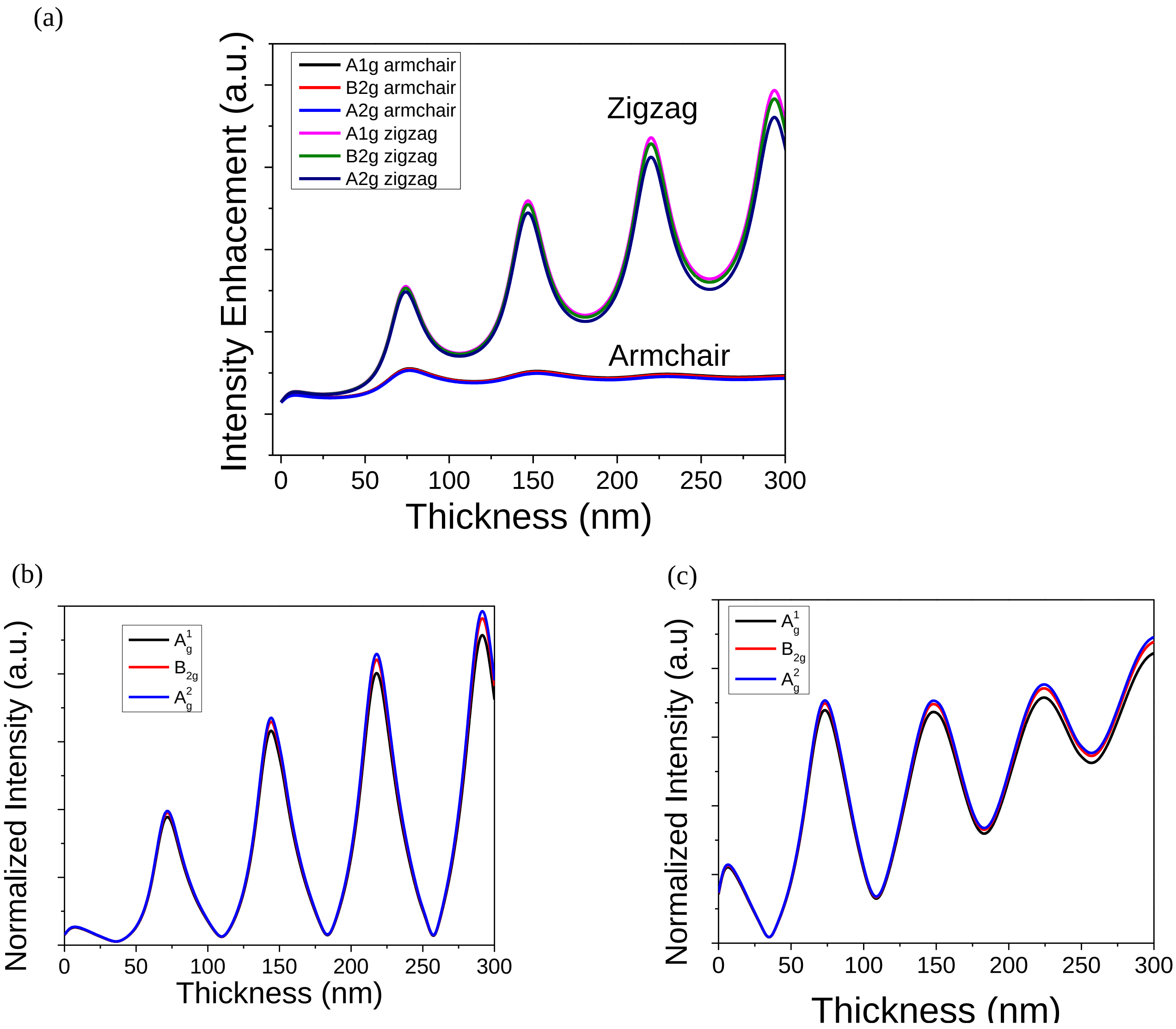

Figure S17. Calculated Raman intensity enhancement (without normalization) of A_g^1, B_{2g}, and A_g^2 Raman modes for zigzag and armchair directions. The normalized Raman intensity enhancement for (b) zigzag and (c) armchair directions are plotted.

b. Thickness and etching rate estimation.

It is possible to estimate the BP thickness using the interference model by fitting the measured intensities of each Raman mode to the calculated enhancement model. In doing so, the boundary conditions should be identified, which are the initial thickness of BP sheet and the SiO_2 thickness. For the flake in figure 2, the thickness is around 35nm, confirmed by AFM measurements, and the SiO_2 thickness is around 280nm. For black phosphorus sheets with thicknesses ranging between 0-40nm, the intensity enhancement profile exhibit negligible differences for zigzag and armchair directions, as illustrated in figure 4. Accordingly, the thickness can be obtained at different time intervals by fitting the normalized intensity profile to the interference model profile described above. Figure S12a, S12b, and S12c show the thickness vs. time calculated for sites A, B, and C on the flake highlighted in figure 3 for A_g^1, B_{2g}, and A_g^2, respectively. In figure 4b of the paper, we plotted the average thickness which is obtained from the S12 figures. The estimated etching rate shown in figure 4c is taken as the derivative of the average thickness.

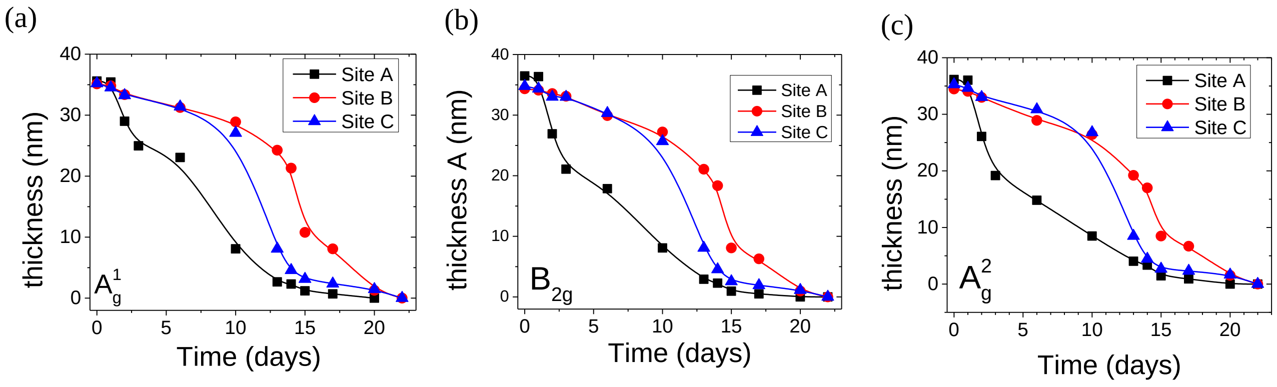

Figure S18. Calculated thickness vs. time for sites A, B, and C considering the Raman intensity of (a) A_g^1 (b) B_{2g}, and (c) A_g^2 Raman modes.

7- Effect of liquid interfaces on the Raman intensity modulation.

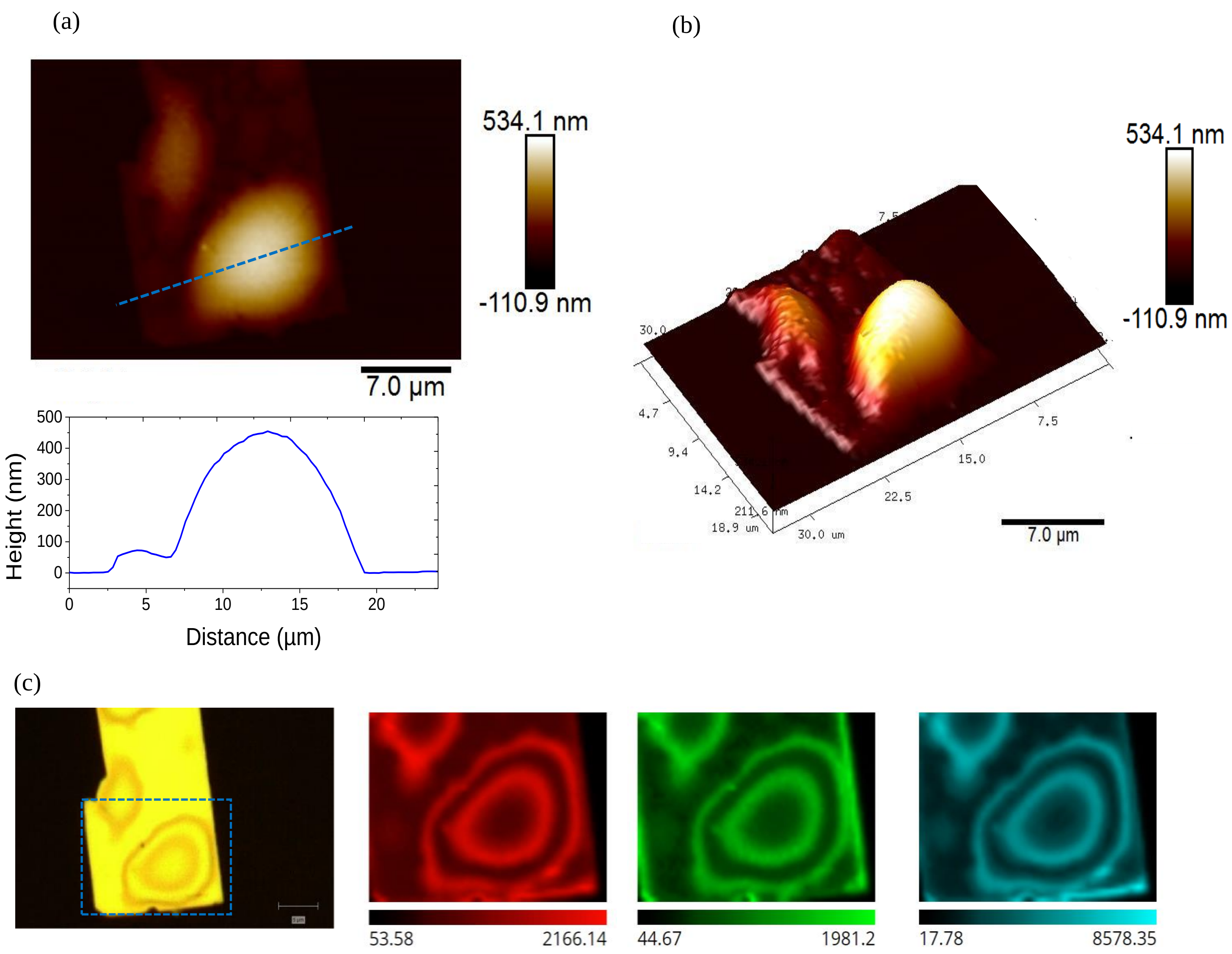

Figure S20. (a) AFM map and (b) 3D overview of a black phosphorus thin film flake. The dashed line in the AFM map illustrates the height profile of the dominant bubble plotted underneath the AFM image. (c) Raman intensity maps of A_g^1 (red), B_{2g} (green), and A_g^2 (cyan) Raman modes of black phosphorus thin film flake after AFM measurements.

(a)

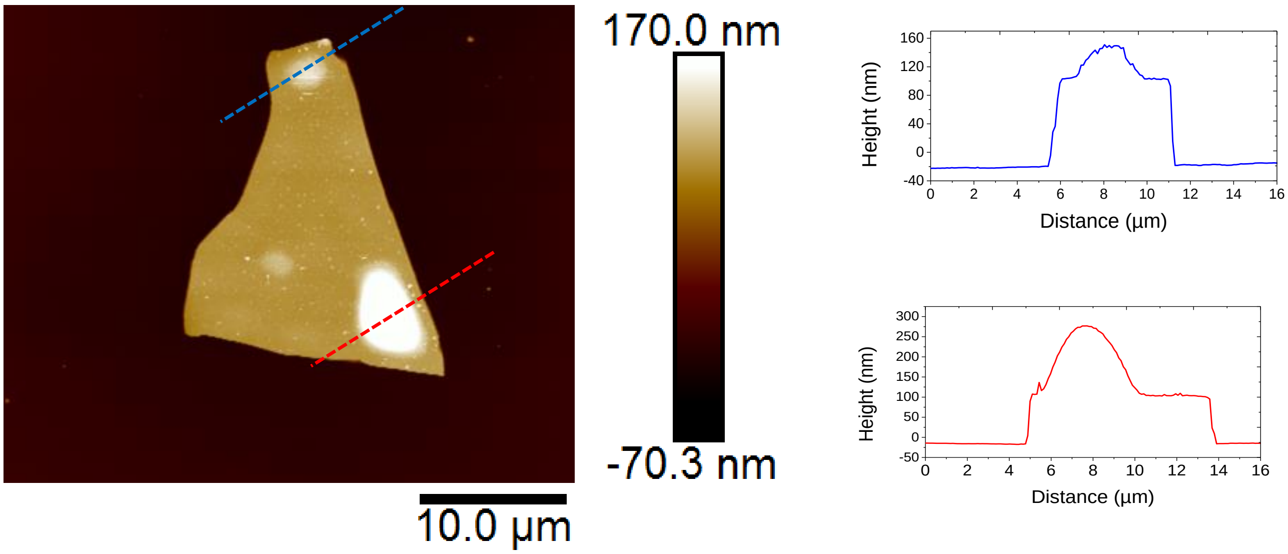

(b)

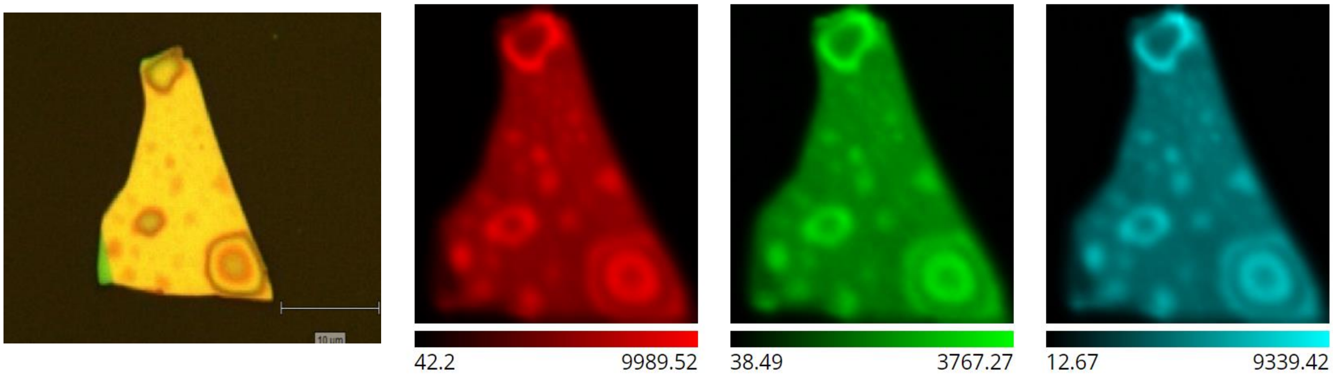

Figure S21. (a) AFM image of a black phosphorus thin film flake. The blue and red dashed lines in the AFM image illustrate the height profile of the upper and bottom bubbles, respectively. (c) Raman intensity maps of A_g^1 (red), B_{2g} (green), and A_g^2 (cyan) Raman modes of black phosphorus thin film flake after AFM measurements.

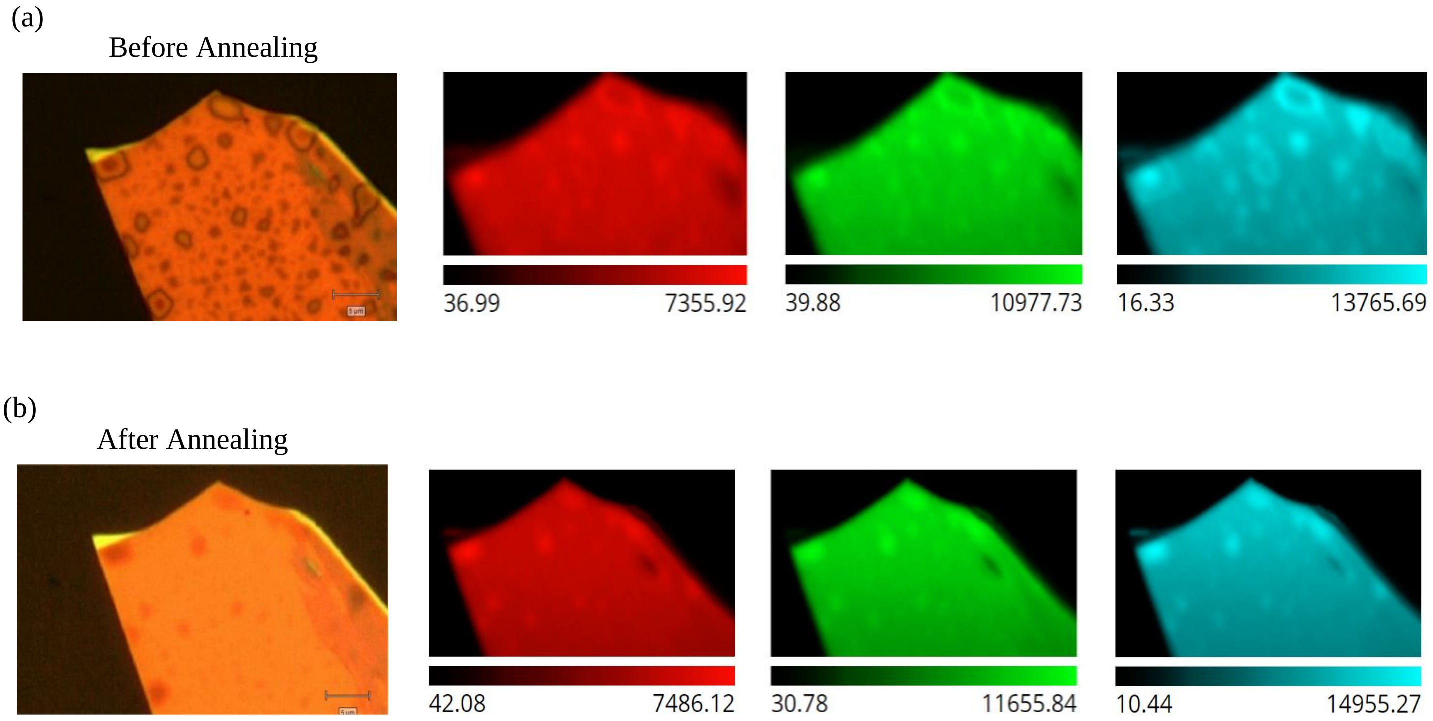

Figure S22. Raman intensity maps of degraded thin film black phosphorus flake (a) before annealing and (b)directly after annealing in vacuum for A_g^1 (red), B_{2g} (green), and A_g^2 (cyan). The optical image of the flake shows a clean surface after annealing due to removal of liquid interfaces.

References:

[1] Favron, A., *et al.*, "Photooxidation and quantum confinement effects in exfoliated black phosphorus," *Nature materials,* vol. 14, pp. 826-832 (2015).

[2] Guo, Z., *et al.*, "From black phosphorus to phosphorene: basic solvent exfoliation, evolution of Raman scattering, and applications to ultrafast photonics," *Advanced Functional Materials*, vol. 25, pp. 6996-7002 (2015).

[3] Asahina, H. and Morita, A., "Band structure and optical properties of black phosphorus," *Journal of Physics C: Solid State Physics*, vol. 17, p. 1839 (1984).

[4] Kim, J., *et al.*, "Anomalous polarization dependence of Raman scattering and crystallographic orientation of black phosphorus," *Nanoscale*, vol. 7, pp. 18708-18715 (2015).

[5] Yoon, D., *et al.*, "Interference effect on Raman spectrum of graphene on SiO 2/Si," *Physical Review B*, vol. 80, p. 125422 (2009).
